# Cyclovirobuxine D, a cardiovascular drug from traditional Chinese medicine, alleviates inflammatory and neuropathic pain mainly *via* inhibition of voltage-gated Ca_v_3.2 channels

**DOI:** 10.3389/fphar.2022.1081697

**Published:** 2022-12-21

**Authors:** Deyuan Su, Ye Gong, Songyu Li, Jian Yang, Yin Nian

**Affiliations:** ^1^ Key Laboratory of Animal Models and Human Disease Mechanisms/Key Laboratory of Bioactive Peptides of Yunnan Province, Ion Channel Research and Drug Development Center, Kunming Institute of Zoology, Chinese Academy of Sciences, Kunming, Yunnan, China; ^2^ State Key Laboratory of Phytochemistry and Plant Resources in West China, Kunming Institute of Botany, Chinese Academy of Sciences, Kunming, Yunnan, China; ^3^ University of Chinese Academy of Sciences, Beijing, China; ^4^ Department of Biological Sciences, Columbia University, New York, NY, United States

**Keywords:** Cyclovirobuxine D, Ca_v_3.2, Ca_v_2.2, *Buxus microphylla*, analgesic effects

## Abstract

Cyclovirobuxine D (CVB-D), the main active constituent of traditional Chinese medicine *Buxus microphylla*, was developed as a safe and effective cardiovascular drug in China. *B. microphylla* has also been used to relieve various pain symptoms for centuries. In this study, we examined and uncovered strong and persistent analgesic effects of cyclovirobuxine D against several mouse models of pain, including carrageenan- and CFA-induced inflammatory pain and paclitaxel-mediated neuropathic hypersensitivity. Cyclovirobuxine D shows comparable analgesic effects by intraplantar or intraperitoneal administration. Cyclovirobuxine D potently inhibits voltage-gated Ca_v_2.2 and Ca_v_3.2 channels but has negligible effects on a diverse group of nociceptive ion channels distributed in primary afferent neurons, including Na_v_1.7, Na_v_1.8, TRPV1, TPRA1, TRPM8, ASIC3, P_2_X_2_ and P_2_X_4_. Moreover, inhibition of Ca_v_3.2, rather than Ca_v_2.2, plays a dominant role in attenuating the excitability of isolated dorsal root ganglion neurons and pain relieving effects of cyclovirobuxine D. Our work reveals that a currently in-use cardiovascular drug has strong analgesic effects mainly *via* blockade of Ca_v_3.2 and provides a compelling rationale and foundation for conducting clinical studies to repurpose cyclovirobuxine D in pain management.

## 1 Introduction

Pain is a major health and socioeconomic burden worldwide, and there has been intensive efforts to develop novel therapeutics for pain treatment and management (Yekkirala et al., 2017; Cohen et al., 2021). Commonly used pain medicines include non-steroidal anti-inflammatory drugs (NSAIDs), amine reuptake inhibitors, antiepileptic drugs and opioids (Laev and Salakhutdinov, 2021; Obeng et al., 2021). Notwithstanding their effectiveness and wide use, these drugs have varying and sometimes inadequate efficacy. When excessively used, they cause deleterious side effects such as gastrointestinal bleeding, anxiety, depressive disorder and addition (Laev and Salakhutdinov, 2021; Obeng et al., 2021). Therefore, there is still a need for the development of novel analgesics.

Many currently used pain medicines are either natural product derivatives from salicylic acid (Aspirin) (Sinniah et al., 2021), morphine (Methadone) (Kreutzwiser and Tawfic, 2020) and *ω*-conotoxin (Ziconotide) (Yang et al., 2019) or developed by repurposing of drugs initially designed for depression and epilepsy (gabapentin (Wiffen et al., 2017) and amitriptyline (Moore et al., 2015)). In this context, further exploration of natural products, in particular clinically acknowledged ones, may substantially accelerate the process of novel analgesics development.

**FIGURE 1:**
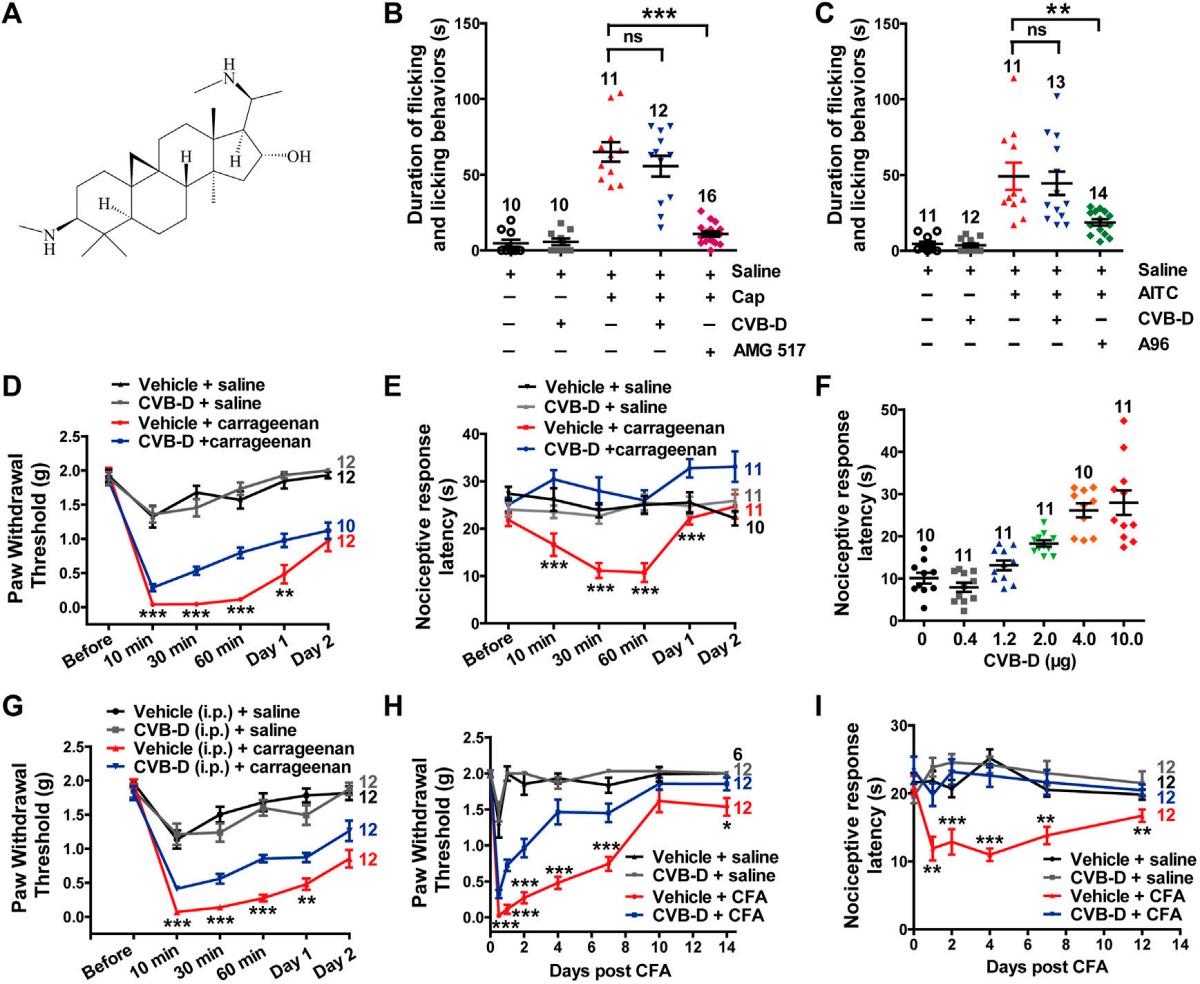
Effect of CVB-D on acute pain and inflammatory hypersensitivity in mouse models. **(A)** Chemical structure of CVB-D. **(B)** Effect of CVB-D on capsaicin (Cap)-induced acute pain. Saline (black) was used as a control for Cap (red), and vehicle (gray) was used as a control for CVB-D (blue). AMG517 (3 mg/kg) treatment was marked as purple. Time of mice spent on licking or lifting of the injected hindpaws was counted within 5 min after i. pl. injection of saline for Saline (black) group, CVB-D for vehicle (gray) group, and Cap for the rest of groups. **(C)** Effect of CVB-D on AITC-induced acute pain. Saline (black) was used as a control for AITC (red), and vehicle (gray) was used as a control for CVB-D (blue). A-967079 (A96) treatment was marked as green. Animal behaviors were recorded using the same method as in Figure 1B. **(D)** Effect of CVB-D (i.pl. injection) on carrageenan-induced mechanical hypersensitivity. PWT was measured with von Frey filaments at the indicated times after i. pl. injection of saline or carrageenan. Vehicle-saline (black) was used as a control for vehicle-carrageenan (red). CVB-D-saline (gray) was used as a control for CVB-D-carrageenan (blue). Statistical analysis was performed between the groups of CVB-D-carrageenan and vehicle-carrageenan. **(E)** Effect of CVB-D (i.pl. injection) on carrageenan-induced thermal hypersensitivity. Same grouping as in Figure 1D. PWL was measured using the hot plate assay at the indicated times after i. pl. injection of saline or carrageenan. Statistical analysis was performed between the groups of CVB-D-carrageenan and vehicle-carrageenan. **(F)** Dose-effect of CVB-D’s (i.pl. injection) suppression on carrageenan-induced thermal hypersensitivity. Each scatter plot shows the PWL of animals received different dosage of CVB-D at 30 min after carrageenan injection. **(G)** Effect of CVB-D (i.p. injection) on carrageenan-induced mechanical hypersensitivity. Same experimental procedures as in Figure 1D, except that CVB-D and its corresponding vehicle were intraperitoneally injected 30 min prior to the i. pl. injection of carrageenan. Statistical analysis was performed between the groups of CVB-D-carrageenan and vehicle-carrageenan. **(H)** Effect of CVB-D (i.pl. injection) on CFA-induced mechanical hypersensitivity. PWT was measured with von Frey filaments within a 2-week period after i. pl. injection of saline or CFA. Vehicle-saline (black) was used as a control for vehicle-CFA (red). CVB-d-saline (gray) was used as a control for CVB-D-CFA (blue). Statistical analysis was performed between the groups of CVB-D-CFA and CFA-vehicle. **(I)** Effect of CVB-D (i.pl. injection) on CFA-induced thermal hypersensitivity. Same grouping as same in Figure 1H. PWL was measured using the hot plate assay at the indicated days after i. pl. administration of saline or CFA. Statistical analysis was performed between the groups of CVB-D-CFA and CFA-vehicle. Data information: The number in each graph indicates the number of mice used in each experiment. Statistical significance was evaluated using two-tailed *t*-test (for two-group comparisons), with *p < 0.05, **p < 0.01, and ***p < 0.001; ns indicates no significance. The exact t, F, and *p*-values are indicated in Appendix Table S1. All the data are presented as mean ± SEM.

*Buxus microphylla* var. Sinica (Chinese Boxwood) is a traditional Chinese medicine (TCM) widely used in cardiovascular disease management for centuries (Bailly and Zhang, 2020). Chinese investigators identified the triterpene alkaloid cyclovirobuxine D (CVB-D) (Figure 1A) as the main active ingredient of *B. microphylla* (Wang and Wang, 1979; Liang et al., 1981). Subsequently, this alkaloid was developed as the active component of Huangyangning dispersible tablets, a well-known drug approved by the China Food and Drug Administration (CFDA) in 2009 for patients with coronary heart disease, angina pectoris, arrhythmias, and even heart failure (Ke et al., 2016; Chinese Pharmacopoeia Commission, 2020). CVB-D has also been reported to hold beneficial potentials on other human diseases, such as tumor (Zeng et al., 2021), ischemic stroke (Ao et al., 2019), and dengue (Wang et al., 2021). Traditionally, *B. microphylla* has also been used for various pain symptoms, including stomachache, toothache, and rheumatic joint pain (Chinese Materia Medica Commission, 1999). However, there is a dearth of knowledge on the molecular mechanisms of this analgesic effect.

Because CVB-D is a major compound of *B. microphylla* and is a clinically used drug, we investigated its analgesic potential and the underlying molecular mechanisms. We examined the effect of CVB-D on several mouse models of pain and a diverse group of ion channels that are expressed in nociceptive primary afferent neurons and known to participate in nociception. These channels include low-voltage-activated (LVA) calcium channels (Ca_v_3.1, Ca_v_3.2 and Ca_v_3.3), high-voltage-activated (HVA) calcium channels (Ca_v_2.2), voltage-gated sodium channels (Na_v_1.7 and Na_v_1.8), transient receptor potential (TRP) channels (TRPV1, TPRA1 and TRPM8), acid-sensing ion channels (ASIC3), and purinergic channels (P_2_X_2_ and P_2_X_4_) (Zamponi et al., 2015; Zamponi, 2016; Yekkirala et al., 2017). Our study shows that intraplantar or intraperitoneal administration of CVB-D significantly alleviates inflammatory and neuropathic pain in mice mainly through inhibition of Ca_v_3.2, an essential target in modern analgesic drug development (Zamponi et al., 2015; Zamponi, 2016; Yekkirala et al., 2017).

## 2 Materials and methods

### 2.1 Chemicals

Cyclovirobuxine D (CVB-D, HY-N0107), Capsaicin (HY-10448) and Paclitaxel (HY-B0015) were purchased from MedChem Express. Allyl isothiocyanate (AITC, 115342) was obtained from Ai Keda Chemical Technology Co. AMG 517 (S7115) was purchased from Selleckchem. A-967079 (B7702) and ATP disodium salt (B3304) were obtained from APExBIO. Carrageenan (S51730) was purchased from Shanghai Yuanye Bio-Technology Co. Ltd. l-cysteine (A600132) was obtained from Sangon Biotech. Z944 hydrochloride (SML2635), SNX-111 (C1182), (±)-Menthol (63670-100G-F) (simply called menthol in this study), CsCH_3_SO_3_ (C1426), Mg-ATP (A9187), CsOH (232041), and Complete Freund’s adjuvant (CFA, F5881) were purchased from Sigma-Aldrich. Other chemicals used in electrophysiology were purchased from Sangon Biotech, including NaCl (A501218), KCl (A501159), CaCl_2_ (A501330), MgCl_2_ (A100288), HEPES (A100511), glucose (A501991), TEA-Cl (A500933), TEA-OH (A501767), NaOH (A100583), KOH (A610441), CsCl (A620054), EGTA (A600077), BaCl_2_ (A602020), K-gluconate (A507810).

### 2.2 Animals and ethics

C57BL/6J mice weighing 18–22 g and aged 6–8 weeks were purchased from SKbex Biotechnology Co., Ltd. and maintained in the animal services facility of Kunming Institute of Zoology. Mice of both genders were randomly divided into 6 to 24 animals per group. We used half male and half female mice for groups with an even animal number, and groups with an odd animal number contained one more mouse with the different gender. The mice were maintained at a constant temperature (22 °C) and humidity (55%) with a 12 h light-dark cycle. Animal husbandry was performed exclusively by the experimenter for the entire duration of the animals’ stay at the housing facility. Lab coats and gloves were changed between cohorts. The housing room was used exclusively for this study and was open to only the experimenter. The mice were acclimatized to the testing facility for at least 1 week before the experiments. Each animal was housed in a single cage to prevent fighting and to freely consume food and water.

All the procedures and care and handling of the animals were approved by the Animal Care and Use Committee at Kunming Institute of Zoology, Chinese Academy of Sciences (IACUC-RE-2022-07-002), and the principles of laboratory animal care (NIH publication No. 86–23, revised 1985) were followed. All animal experiments were performed at room temperature (approximately 23°C).

### 2.3 Acute models of pain

Before measurement of capsaicin- or AITC-induced acute pain, mice were acclimated to a Plexiglas chamber for at least 30 min. Based on toxicological study of CVB-D (Yu et al., 2008), saturated concentration of CVB-D (10.07 μg, 10 μL/paw) was first freshly prepared in 5% ethanol saline solution (vehicle) and administrated into mice hindpaws by intraplantar (i.pl.) injection using micro-syringe with a 30-gauge needle. The same volume of vehicle was administrated by i. pl. injection as control. Dose-dependent relationship of CVB-D on animal model was further conducted and described in next section. Thirty minutes after CVB-D or vehicle injection, freshly prepared 100 μM capsaicin saline solution (10 μL/paw), 1 mM AITC saline solution (20 μL/paw), or control saline was administrated into the plantar surface of the injected hindpaws. The mice were immediately returned to the Plexiglas chamber and recorded pain behaviors using a digital video camera. Time of mice spent on licking or lifting of the injected hindpaws was counted within 5 min after acute pain induction. In several experiments, a selective TRPV1 inhibitor A-967079 (10 mg/kg) or a specific TRPA1 blocker AMG517 (3 mg/kg) was administrated by intraperitoneal (i.p.) injection 1 h prior to the i. pl. application of capsaicin or AITC.

Capsaicin-induced thermal hypersensitivity was conducted as described previously (DuBreuil et al., 2021). Mice firstly received a single i. pl. injection of a vehicle (10 μL/paw, 1% DMSO in saline for SNX-111 and Z944; 5% ethanol in saline for CVB-D), SNX-111 (10 μL/paw, 53 ng), CVB-D (10 μL/paw, 10.07 μg) or Z944 (10 μL/paw, 3.84 μg). Thirty minutes later mice received freshly prepared 100 μM capsaicin saline solution (10 μL/paw) by i. pl. injection. Thermal hypersensitivity of mice was evaluated by the hot plate assay (Beijing Zhongshidichuang Science and Technology Development Co., Ltd., ZS-CTE). Briefly, mice were placed on a stainless-steel plate with a temperature setting of 52°C (60 s cutoff time), and the paw withdrawal latency (PWL) for the mice to exhibit a painful response (licking hindpaw or jumping from the plate) was recorded. Behavioral assessments were conducted at 0 min, 15 min and 30 min after thermal hypersensitivity induction.

### 2.4 Inflammatory models of pain

Peripheral inflammatory painmodels were generated by i. pl. injection of carrageenan or CFA into the hindpaws of mice. We used 3% carrageenan in saline to induce sub-acute inflammation as described previously (Helyes et al., 2009; Yen et al., 2009). Mice firstly received a single i. pl. or i. p. injection of a vehicle (10 μL/paw, same as in 2.3), SNX-111 (10 μL/paw, 53 ng), CVB-D (10 μL/paw, 10.07 μg for i. pl. injection; 5 mg/kg for i. p. administration), Z944 (10 μL/paw, 3.84 μg for i. pl. injection; 3 mg/kg for i. p. administration), and a combination (by i. pl.) of SNX-111 and CVB-D or Z944. For dose-dependent relationship study of CVB-D, mice received a single i. pl. injection of CVB-D (10 μL/paw) with the concentration from 0.4 to 10.07 μg. Thirty minutes later, the mice were divided into two groups: those received another i. pl. injection of saline (50 μL/paw) and carrageenan (50 μL/paw), respectively. Behavioral assessments (von Frey test or hot plate assay) were conducted at 30 min before, and 10 min, 30 min, 60 min, 1 day, and 2 days after hypersensitivity induction.

In CFA model, mice firstly received a single i. pl. injection of a vehicle (10 μL/paw, same as in 2.3), CVB-D (10 μL/paw, 10.07 μg) or Z944 (10 μL/paw, 3.84 μg). Thirty minutes later, 20 μL of CFA (1:1 emulsion of saline) or saline was administrated by i. pl. injection. The time of receiving such treatments was set as Day 0. Mechanical and thermal hypersensitivity were studied within a 2-week period after CFA injection. Mechanical hypersensitivity was examined on Day 0, Day 1, Day 2, Day 4, Day 7, Day 10, and Day 14, and thermal hypersensitivity was evaluated on Day 0, Day 1, Day 2, Day 4, Day 7, and Day 12.

For assessment of mechanical hypersensitivity, mice were placed in a plastic cage with a wire mesh bottom and were allowed to acclimate until cage exploration and major grooming activities ceased. 50% mechanical paw withdrawal threshold (PWT) was assessed with von Frey filaments (North Coast Medical Inc., NC12775) using the up-down method (Chaplan et al., 1994). Thermal hypersensitivity was evaluated using the hot plate assay as described in Section 2.3.

### 2.5 l-cysteine-induced pain model

For l-cysteine-induced hypersensitivity model, mice were given l-cysteine based on the protocol as described previously (Todorovic et al., 2001). In brief, mice firstly received a single i. pl. injection of a vehicle (10 μL/paw, same as in 2.3), CVB-D (10 μL/paw, 10.07 μg) or Z944 (10 μL/paw, 3.84 μg). Thirty minutes later, the mice received l-cysteine (1 μg/paw, 20 μL) or saline by i. pl. injection. Behavioral assessments (von Frey test or hot plate assay) were conducted at 10 min after hypersensitivity induction using the same approaches as described in Section 2.3 and Section 2.4.

### 2.6 Paclitaxel-induced neuropathic pain model

Mice were given paclitaxel based on the protocol as described previously (Abed et al., 2017; Li et al., 2017; Caillaud et al., 2021). In brief, mice received 4 mg/kg i. p. administration of pharmaceutical-grade paclitaxel every other day for a total of four injections (days 0, 2, 4, and 6), resulting in a final cumulative dose of 16 mg/kg. Thirty minutes before the first administration of paclitaxel, mice received a single i. pl. injection of a vehicle (10 μL/paw, same as in 2.3), CVB-D (10 μL/paw, 10.07 μg) or Z944 (10 μL/paw, 3.84 μg). Control animals received an equivalent volume of saline only. Thermal hypersensitivity was studied using the same method as described in Section 2.3. The baseline for thermal hypersensitivity was evaluated 30 min before CVB-D or Z944 application (Day 0). The checkpoints were Day 0, Day 4, Day 7, Day 10, and Day 14.

Blinding was not carried out for above-mentioned mouse models, as the tester also conducted the modeling and all animal husbandry. In addition, the effects of these models are obvious when handling the animals and performing testing.

### 2.7 Primary dorsal root ganglion neuron culture

Inhalation anaesthesia was firstly applied to adult mice. Then dorsal root ganglia were dissected, rinsed with Hank’s buffer (Gibco, 13150016), and digested in the same buffer containing 1.5 mg/ml collagenase P (Roche Diagnostics, 11213865001) for 25–45 min at 37°C. Partially digested tissues were centrifuged at 200 × g for 3 min, and the pellets were resuspended in 0.25% trypsin-EDTA (Gibco, 25200056) and digested for an additional 5 min at 37°C. The digested ganglia were spun down, resuspended, and triturated with plastic pipette tips to release the neurons. The cells were filtered through a 70-μm cell strainer (Biologix, 15–1070), plated into 24-well plates, then aliquot and cultured in poly-d-lysine-treated 35 mm dishes containing DMEM/F-12 (Gibco, 11320033) supplemented with GlutaMAX (Gibco, 35050061) and 10% fetal bovine serum (Gibco, 10100). Electrophysiological experiments were performed after 2 h of culture.

### 2.8 HEK 293T cell culture and transfection

HEK 293T cells (American Type Culture Collection (ATCC) were grown in DMEM (Biological Industries, 01-055-1A) plus 10% fetal bovine serum (Gibco, 10100) and 1% penicillin (100 U/ml)/streptomycin (0.1 mg/ml; Biological Industries, 03-031-1B). HEK 293T cells were transiently transfected with pCDNA3.1-human TRPA1 (NM_007332.3), human TRPV1 (AJ277028.1), human TRPM8 (NM_024080.5), rat P_2_X_2_ (NM_053656.3), human P_2_X_4_ (NM_002560.3), rat ASIC3 (NM_173135.1), rat Na_v_1.7 (NM_133289.1), human Na_v_1.8 (NM_006514.3), human Ca_v_3.1 (NM_198387.3), human Ca_v_3.2 (NM_021098.3), human Ca_v_3.3 (NM_021096.4) and human Ca_v_2.2 (NM_000718.4) (with rat β3 (NM_012828.3) and rabbit α2δ (NM_001082276.1) subunits), together with pEGFPN1 (Addgene, 6085-1) plasmids using LipoD293 *in vitro* DNA Transfection Reagent (SignaGen Laboratories, SL100668) and used within 48 h.

### 2.9 Electrophysiology

The patch clamp amplifier Axopatch 200B (Axon, United States) and Double IPA (Sutter Instrument, United States), which is an integrated patch clamp amplifier with data acquisition system, were used for cell electrical signal amplification. A digital-to-analog converter Digidata 1440A (Axon, United States) was used for digital electrical signal conversion when Axopatch 200B was used. Currents were low-pass filtered at 2 kHz and sampled at 10 kHz. Recordings were included for analysis only if access resistance began below 25 MΩ and did not change by more than 30% during the recording period. pCLAMP 10 software (Molecular Devices, United States) and SutterPatch2.1 software (Sutter Instrument) were used for data acquisition and analysis. All experiments were performed at room temperature (approximately 23°C). For patch-clamp recordings, pipettes were fabricated from borosilicate glass (World Precision Instruments, PG52151-4) using a micropipette puller (P-1000, Sutter Instrument), and were fire-polished to resistances of 4–6 MΩ for whole-cell recording. All the experiments where comparisons in different conditions were made were conducted in a double-blind way.

For T-type calcium channels (Ca_v_3.1-3.3) current measurements, the extracellular solution contained (in mM) 142 CsCl, 1 MgCl_2_, 2 CaCl_2_, 10 Glucose and 10 HEPES (pH 7.4 adjusted with CsOH). The intracellular solution contained (in mM) 142 CsCl, 2 MgCl_2_, 11 EGTA, 5 Na_2_-ATP, 10 HEPES (pH 7.4 adjusted with CsOH). Peak currents of Ca_v_3.1 and Ca_v_3.2 were elicited by 150-ms depolarizations to −40 mV at 4-s intervals from a holding potential (HP) of −100 mV. For recording peak current of Ca_v_3.3, the stimulus time was set to 400 ms. For studying state-dependent of Ca_v_3.2, the HP was set to −100 mV and −75 mV, respectively. For studying use-dependent of Ca_v_3.2, the stimulus frequencies were set to 0.1 Hz and 1 Hz, respectively. I-V curves of Ca_v_3.1 and Ca_v_3.2 were evoked by 150-ms depolarizations from −80 mV to + 60 mV in 10-mV increments with a 4-s interval from a HP of −100 mV. For recording I-V curve of Ca_v_3.3, the stimulus time was set to 400 ms.

Voltage-dependent activation of Ca_v_3.2 was stimulated by applying a series of 150-ms step depolarizations from −80 to +40 mV in 5-mV increments with a 4-s interval from a HP of −100 mV. The peak current at each voltage was measured and the corresponding conductance G) was calculated using the equation: G = I/(V − V_rev_), where V is the test voltage and V_rev_ was calculated by linear extrapolation of peak currents with depolarization potentials from 10 to 40 mV. Normalized G was then plotted against voltage, and activation curves were obtained by Boltzmann fitting: G/Gmax = 1/{1 + exp [(V_1/2_ − V)/k]}, where Gmax is the maximal conductance, V_1/2_ is the potential of half-maximal activation, and k is the slope factor.

Inactivation of Ca_v_3.2 was studied by comparing the current amplitude (P1) elicited by a voltage step to −40 mV (10 ms) in the absence of fast inactivation to a second step (P2) following a series of 150-ms inactivating steps ranging from −100 to −30 mV in 5-mV increments with a 4-s interval from a HP of −110 mV. P1 was followed by a 1-s recovery step to −110 mV to remove any inactivation induced by P1. The P2/P1 ratio was used as a measure of the fraction of available channels. Normalized residual current was plotted against the voltage of the conditioning pulses. Inactivation curves were fitted with Boltzmann function in the form of I/Imax = 1/{1 + exp [(V_1/2_ − V)/k]}.

For N-type calcium channel (Ca_v_2.2) current measurements, the extracellular solution contained (in mM) 105 CsCl, 40 TEA-Cl, 2 BaCl_2_, 1 MgCl_2_, 10 d-glucose, and 10 HEPES (pH 7.4 adjusted with CsOH). The intracellular solution contained (in mM) 130 CsCH_3_SO_3_, 10 TEA-Cl, 10 EGTA, 10 HEPES, 5 MgCl_2_, and 5 Na_2_-ATP (pH 7.4 adjusted with CsOH). Peak currents of Ca_v_2.2 were elicited by 500-ms depolarizations to 0 mV at 4-s intervals from a HP of −80 mV. For the state-dependent studies, the HP was set to −80 and −60 mV, respectively. I-V curve of Ca_v_2.2 was evoked by 500-ms depolarizations from −60 mV to +70 mV in 10-mV increments with a 4-s interval from a HP of −80 mV.

For TRP channel recordings, the extracellular solution contained (in mM) 150 NaCl, 1 MgCl_2_, and 10 HEPES (pH 7.4 adjusted with NaOH). The intracellular solution contained (in mM) 150 NaCl, 1 MgCl_2_, 1 EGTA, and 10 HEPES (pH 7.4 adjusted with NaOH). For ASIC3, P_2_X_2_ and P_2_X_4_ recordings, the extracellular solution was the same as that of TPR channels. The intracellular solution contained (in mM) 140 KCl, 5 EGTA, and 10 HEPES (pH 7.4 adjusted with NaOH). The whole-cell currents of TRPA1, TRPM8 and TRPV1 were elicited by 500-ms voltage ramps from −100 to +100 mV at a frequency of 0.5 Hz with a HP of 0 mV. The whole-cell currents of ASIC3, P_2_X_2_ and P_2_X_4_ were recorded by the gap-free mode with a HP of −60 mV. For Na_v_1.7 and Na_v_1.8 recordings, the internal solution contained (in mM) 135 K-gluconate, 5 KCl, 5 Mg-ATP, 0.5 Na_2_GTP, 5 HEPES, 2 MgCl_2_, 5 EGTA, and 0.5 CaCl_2_ adjusted to pH 7.4 with KOH, and the bath solution contained (in mM) 140 NaCl, 5 KCl, 2 CaCl_2_, 2 MgCl_2_, 10 HEPES, and 10 glucose, adjusted to pH 7.4 with NaOH. Peak currents of Na_v_1.7 and Na_v_1.8 were evoked by 10-ms depolarizations to −30 mV at 5-s intervals from a HP of −110 mV.

For whole-cell recording of dorsal root ganglion (DRG) neurons, 2 hours after being plated, dissociated DRG neurons were perfused with an extracellular solution containing (in mM) 10 BaCl_2_, 152 TEA-Cl, and 10 HEPES adjusted to pH 7.4 with TEA-OH. The recording electrode was filled with a solution containing (in mM) 135 TEA-Cl, 10 EGTA, 40 HEPES, and 2 MgCl_2_ adjusted to pH 7.2 with TEA-OH. LVA calcium channel peak currents were evoked by 250-ms depolarizations to −40 mV at 4-s intervals from a HP of −100 mV. HVA calcium channel peak currents were evoked by 250-ms depolarizations to +10 mV at 4-s intervals from a HP of −80 mV. DRG neurons with diameters ranging 10–30 μm were chosen for recording.

For current-clamp recording of DRG neurons, the internal and bath solutions were the same as those for Na_v_ channels recording. DRG neurons were held at 0 pA, and the threshold current of action potential was evoked using a series of 500-ms depolarizing current injections in 10-pA steps from 0 pA. Only neurons with a resting membrane potential below −40 mV, had stable baseline recordings, and showed evoked spikes over 0 mV were used for further experiments and analysis. Series resistance was compensated to above 75% for the recorded DRG neurons.

For all electrophysiological experiments, extracellular solutions containing CVB-D, Z944, SNX-111, AITC, menthol, capsaicin, or ATP were prepared immediately before the experiments.

### 2.10 Statistics

We used sample/animal sizes that were deemed suitable for statistics and were similar to other studies in the field. Animals were randomly selected and allocated to experimental groups. Data normality was assessed by the Shapiro-Wilk method, and the equality of variances for two or more data groups was determined by F-test or Levene’s test. Statistical significance was evaluated using two-tailed *t*-test (for all two-group comparisons) or one-way analysis of variance (ANOVA) followed by Tukey’s and LSD test (for multi-group comparisons). Data are presented as the mean ± standard error of the mean (SEM), and a *p*-value <0.05 is considered statistically significant, with *p < 0.05, **p < 0.01, and ***p < 0.001. For multi-group comparisons, asterisks, pound signs or ampersand was used respectively, ns indicates no significant difference.

## 3 Results

### 3.1 Cyclovirobuxine D alleviates inflammatory pain

To evaluate the analgesic effect of CVB-D, we first examined its alleviationon acute pain induced by capsaicin (Cap) and AITC, which activate TRPV1 and TRPA1, respectively. Cap or AITC (i.pl. injection) caused robust acute pain responses in mice, manifested as licking or lifting of the injected hindpaws (Figures 1B,C). These responses were largely prevented by prior administration of TRPV1-specific antagonist AMG 517 or selective TRPA1-inhibitor A-967079 (A96) with the *p*-value of *p* < 0.001 and *p* = 0.007 respectively, but not CVB-D (Figures 1B,C *p* = 0.329 for Cap VS. CVB-D; *p* = 0.697 for AITC VS. CVB-D). CVB-D (i.pl. injection) alone did not induce hypersensitive responses (Figures 1B,C, and Supplementary Figure S1A. *p* = 0.529). Thus, CVB-D does not affect TRPA1 or TRPV1-mediated acute pain.

We next examined the effect of CVB-D on two typical mouse inflammatory pain models induced by i. pl. injection of carrageenan and CFA, respectively. In the carrageenan-induced model, there was no noticeble difference in the mechanical hypersensitivity between male and female mice in both control and test groups (Supplementary Figure S1B. *p*-value of each test point is 0.221, 0.551, 0.451, 0.389, 0.495, and 0.492, respectively). Thus, results from both genders were combined for data analysis and statistics. Carrageenan-induced mechanical and thermal hypersensitivity, manifested as a decreased paw withdrawal threshold (PWT) or paw withdrawal latency (PWL), respectively, peaked at 10–30 min, and gradually decayed over 2 days (sub-acute phases). CVB-D (10.07 μg) administrated alone by i. pl. injection did not affect the PWT or PWL in control animals (Figures 1D,E, and Supplementary Figures S1C,D. In Supplementary Figure S1C, *p* = 0.796 for saline VS. vehicle, *p* = 0.578 for saline VS. CVB-D, and *p* = 0.780 for vehicle VS. CVB-D. In Supplementary Figure S1D, *p* = 0.932 for saline VS. vehicle, *p* = 0.390 for saline VS. CVB-D, and *p* = 0.717 for vehicle VS. CVB-D). By contrast, it (i.pl. injection) reduced both acute (10, 30, and 60 min) and sub-acute phases (Day 1) of mechanical hypersensitivity (Figure 1D *p*-value of each test point is 0.300, 0.001, <0.001, <0.001, 0.009, and 0.463, respectively). Strikingly, at this dosage CVB-D prevented the development of thermal hypersensitivity (Figure 1E *p*-value of each test point is 0.121, 0.000, <0.001, <0.001, <0.001, and 0.053, respectively). In the dosage range of 0.4–10.0 μg by i. pl. injection, CVB-D dose-dependently prolonged the PWL measured at 30 min after carrageenan injection, with an EC_50_ of 2.03 ± 0.53 μg (Figure 1F and Supplementary Figure S1E). Administration of CVB-D (5 mg/kg) by i. p. injection also produced obviousanalgesic effect on mechanical hypersensitivity without affecting the baseline of PWT (Figure 1G and Supplementary Figure S1F. *p*-value in Figure 1G of each test point is 0.234, 0.000, 0.000, 0.000, 0.002, and 0.051, respectively. In Supplementary Figure S1F, *p* = 0.596 for saline VS. vehicle, *p* = 0.623 for saline VS. CVB-D, and *p* = 0.106 for vehicle VS. CVB-D). Therefore, both intraplantar and intraperitoneal administration of CVB-D produced clear analgesic effects on carrageenan-induced inflammatory hypersensitivity.

CFA-induced hypersensitivity is another well-studied chronic inflammatory pain model, with hypersensitive responses lasting for approximately 1–2 weeks (Berger et al., 2014; Lin et al., 2016). A single dose of CVB-D (10.07 μg) by i. pl. injection attenuated mechanical hypersensitivity (Figure 1H *p*-value of each test point is < 0.001, <0.001, <0.001, <0.001, <0.001, 0.185, and 0.048, respectively) and largely eliminated thermal hypersensitivity (Figure 1I *p*-value of each test point is 0.383, 0.003, 0.001, <0.001, 0.002, and 0.007, respectively) during 2 weeks after injection of CFA. These long-lasting effects of CVB-D hint that its actions may not only involve the initiation but also the maintenance of CFA-induced hypersensitivity.

These results indicate that in addition to its cardiovascular bioactivities, CVB-D has strong analgesic effect on inflammatory pain hypersensitivity.

### 3.2 Effect of cyclovirobuxine D on recombinant nociceptive ion channels

**FIGURE 2:**
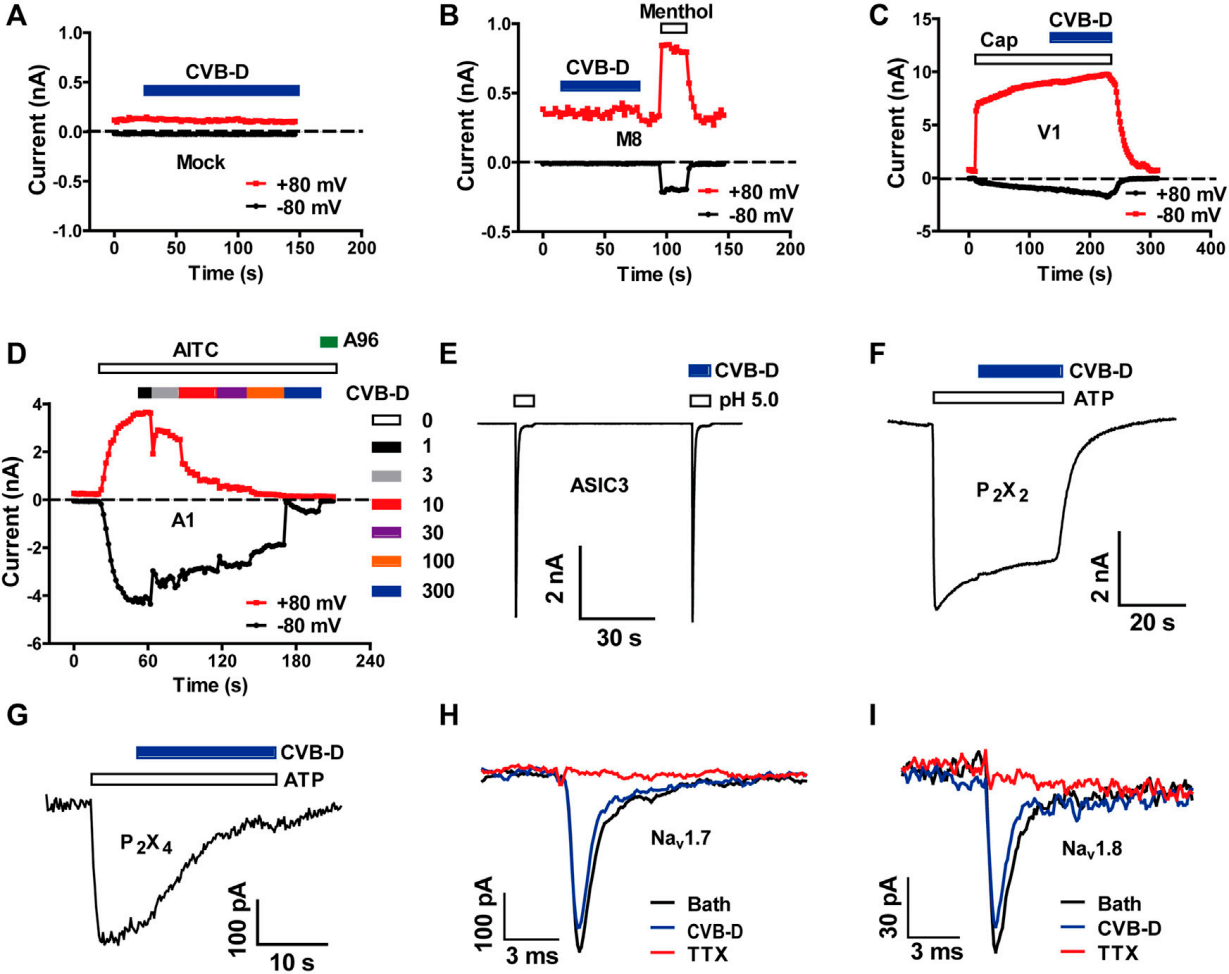
Effect of CVB-D on recombinant nociceptive ion channels. **(A–G)** Representative whole-cell currents in mock transfected HEK 293T cells **(A)** or cells expressing TRPM8 **(B)**, TRPV1 **(C)**, TRPA1 **(D)**, ASIC3 **(E)**, P_2_X_2_ **(F)** and P_2_X_4_ **(G)** in response to 500 μM methol **(B)**, 1 μM capsaicin **(C)**, 100 μM AITC **(D)**, extracellular solution with an acidic pH of 5.0 **(E)**, and 100 μM ATP **(F,G)** in the absence or presence of 30 μM CVB-D (n = 4 for each channel). **(H,I)** Representative whole-cell currents in HEK 293T cells expressing Na_v_1.7 and Na_v_1.8 in the absence or presence of 30 μM CVB-D.

To investigate the underlying molecular targets for analgesia by CVB-D, we firstly examined its effect on several ion channels known to be involved in peripheral nociception. CVB-D (30 μM) did not affect the basal whole-cell current of mock-transfected HEK 293T cells, and did not affect agonist-induced currents in HEK 293T cells expressing TRPM8 and TRPV1, which sense noxious temperature and chemicals (Figures 2A–C, and Supplementary Figure S2A,B) (Yekkirala et al., 2017). CVB-D did not activate TRPA1, another noxious chemical sensing ion channel, but dose-dependently inhibited AITC-induced inward-currents, with an IC_50_ value of 16.03 ± 0.11 μM (Figure 2D and Supplementary Figure S2C,D). Of note, CVB-D has no analgesic effect on AITC-induced acute pain (Figure 1C), indicating that TRPA1 is not an underlying target for CVB-D at the dosages used in this experiment. CVB-D (30 μM) also had little effect on several other ion channels involved in inflammatory hypersensitivity, including acid-sensing ion channel 3 (ASIC3) and P_2_X channels, P_2_X_2_ and P_2_X_4_ (Figures 2E–G and Supplementary Figure S2E) (Yekkirala et al., 2017), and had negligible effect on pain-related Na_v_1.7 and Na_v_1.8 voltage-gated Na^+^ channels (Figures 2H,I) (Hameed, 2019; Xue et al., 2021).

### 3.3 Cyclovirobuxine D inhibits recombinant Ca_v_3.2 and Ca_v_2.2 channels

**FIGURE 3:**
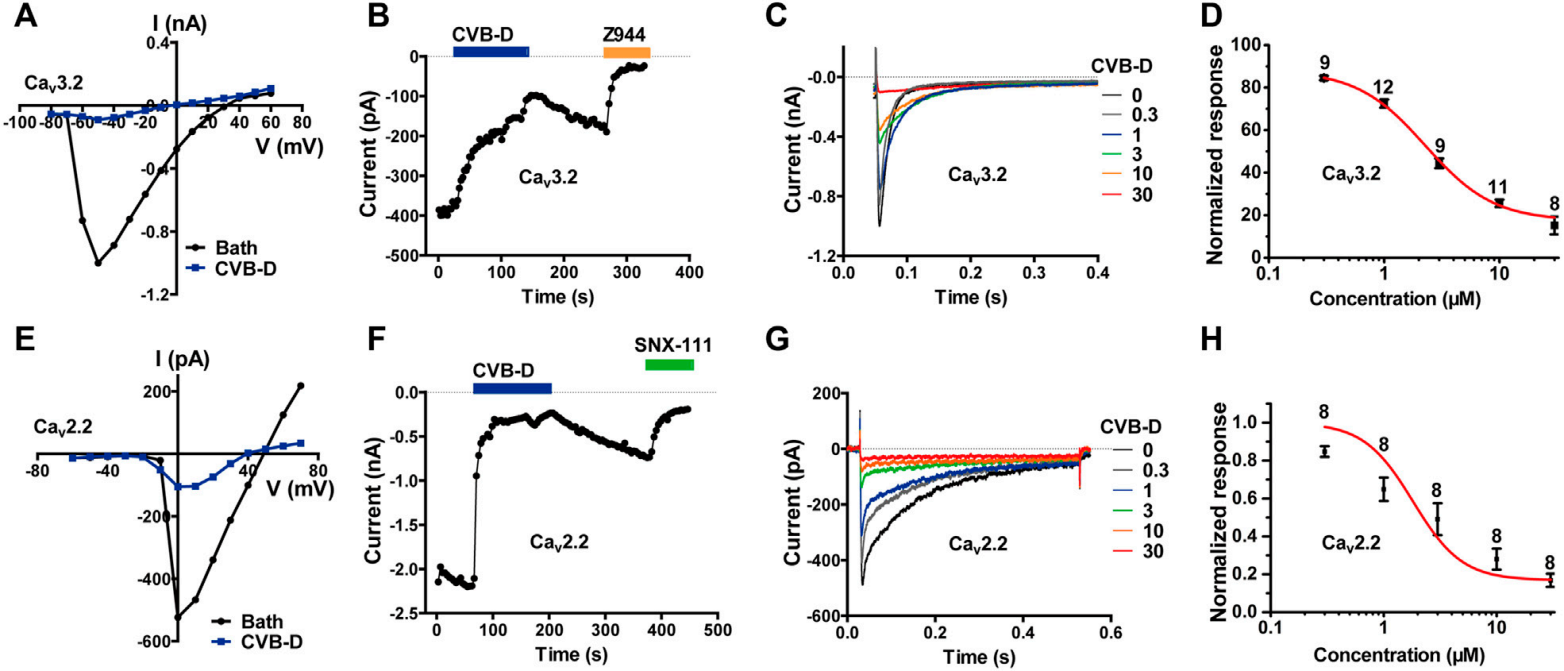
CVB-D-mediated inhibition of recombinant Ca_v_3.2 and Ca_v_2.2 channels expressed in HEK 293T cells. **(A)** Representative current-voltage (I-V) relationship of Ca_v_3.2 in the absence or presence of 30 μM CVB-D. **(B)** Time course of inhibition by CVB-D (30 μM) and subsequent inhibition by Z944 (10 μM) of Ca_v_3.2 peak current. **(C)** Inhibition of Ca_v_3.2 peak current by different concentrations of CVB-D. **(D)** Dose-response relationship of CVB-D inhibition of Ca_v_3.2 peak current. Solid curve represents fit to the Hill equation. Data are presented as mean ± SEM. n = 8–12 for each concentration. **(E)** Representative I-V relationship of Ca_v_2.2 in the absence or presence of 10 μM CVB-D. **(F)** Time course of inhibition by CVB-D (10 μM) and subsequent inhibition by SNX-111 (0.5 μM) of Ca_v_2.2 peak current. **(G)** Inhibition of Ca_v_2.2 peak current by different concentrations of CVB-D. **(H)** Dose-response relationship of CVB-D inhibition of Ca_v_2.2 peak current. Solid curve represents fit to the Hill equation. Data are presented as mean ± SEM. n = 8 for each concentration.

LVA calcium channels, namely T-type calcium channels (TTCCs), consist of Ca_v_3.1-3.3. Among them, Ca_v_3.2 is the dominant isoform of TTCCs that abundantly expressed in nociceptive neurons and has been considered as a promising target for neuropathic, inflammatory, and visceral pain (Jevtovic-Todorovic and Todorovic, 2006; Cai et al., 2021; Hoppanova and Lacinova, 2022). We therefore examined the effect of CVB-D on Ca_v_3 channels. CVB-D at 30 μM robustly inhibited whole-cell current in HEK 293T cells expressing Ca_v_3.2 (Figure 3A). This inhibition was relatively slow and could be partly reversed (Figure 3B). The remaining current was completely blocked by Z944 (10 μM) (Figure 3B), a high affinity TTCCs inhibitor under clinical trial (Lee, 2014; Harding et al., 2021). Dose-response relationship of CVB-D inhibition of Ca_v_3.2 peak current obtained at a HP of −100 mV yields an IC_50_ of 2.28 ± 0.35 μM (Figures 3C,D). CVB-D also dose-dependently inhibited Ca_v_3.1 and Ca_v_3.3 expressed in HEK 293T cells, with IC_50_ values of 2.11 ± 0.09 μM and 2.02 ± 1.29 μM, respectively (Supplementary Figure S3A,B).

Among HVA calcium channels, Ca_v_2.2 (N-type) is a well-established target for antinociceptive drug development (Hoppanova and Lacinova, 2022). CVB-D dose-dependently inhibited Ca_v_2.2 expressed in HEK 293T cells, with an IC_50_ value of 1.82 ± 1.40 μM (Figures 3E–H). Like its inhibition of Ca_v_3.2, CVB-D’s blockade of Ca_v_2.2 was only partially reversible upon washout of CVB-D (Figure 3F), and the residual current could be totally inhibited by SNX-111 (0.5 μM), a selective Ca_v_2.2 blocker that has been clinically used for morphine insensitive pain symptoms (Figure 3F).

These results, together with those above, demonstrate that among a diverse group of peripheral nociceptive ion channels CVB-D mainly inhibits T-type and N-type calcium channels expressed in HEK 293T cells.

### 3.4 Regulation of some other electrophysiological properties of recombinant Ca_v_3.2 and Ca_v_2.2 channels by cyclovirobuxine D

**FIGURE 4:**
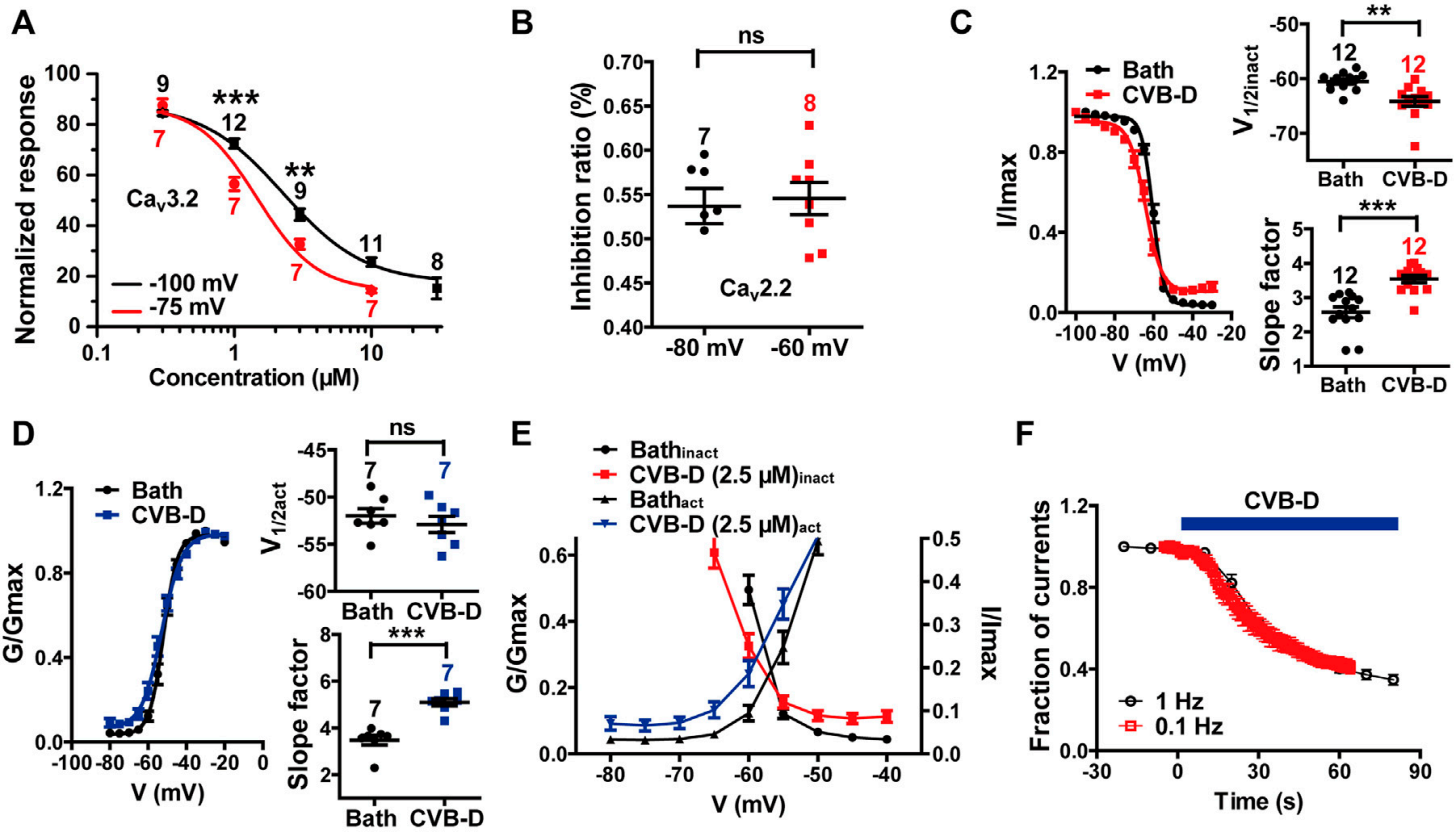
Effect of CVB-D on electrophysiological properties of recombinant Ca_v_3.2 and Ca_v_2.2 channels expressed in HEK 293T cells. **(A)** Dose-response relationship of CVB-D inhibition of Ca_v_3.2 peak current from a HP at −100 mV (black) or −75 mV (red), respectively. Solid curve represents fit to the Hill equation. **(B)** Inhibition by 2.5 μM CVB-D of Ca_v_2.2 at a HP of −80 mV or -60 mV, respectively. **(C)** Effect of CVB-D (2.5 μM) on Ca_v_3.2 inactivation. Left panel is the inactivation curve of Ca_v_3.2 in the presence (red) or absence of CVB-D (black). Solid curve represents fit to the Boltzmann equation. Right upper panel is the comparison of the half-inactivation potential (V_1/2_) in the presence (red) or absence of CVB-D (black). Right lower panel is the comparison of the slope factor in the presence (red) or absence of CVB-D (black). **(D)** Effect of CVB-D (2.5 μM) on Ca_v_3.2 activation. Left panel is the activation curve of Ca_v_3.2 in the presence (blue) or absence of CVB-D (black). Solid curve represents fit to the Boltzmann equation. Right upper panel is the comparison of the half-activation potential (V_1/2_) in the presence (blue) or absence of CVB-D (black). Right lower panel is the comparison of the slope factor in the presence (blue) or absence of CVB-D (black). **(E)** Comparison of Ca_v_3.2 window currents in the presence (red for inactivation curve, blue for activation curve) or absence of CVB-D (black). **(F)** Effect of CVB-D on Ca_v_3.2 stimulated at a frequency of 0.1 or 1 Hz. Data information: The number in each graph indicated the number of cells used in each experiment. Statistical significance was evaluated using two-tailed *t*-test (for two-group comparisons), with *p < 0.05, **p < 0.01, and ***p < 0.001; ns indicates no significance. The exact t, F, and *p*-values are indicated in Appendix Table S1. All the data are presented as mean ± SEM.

We also examined the effect of CVB-D on some other electrophysiological properties of Ca_v_3.2 and Ca_v_2.2 expressed in HEK 293T cells. Because Ca_v_3.2 plays a major role in the analgesic effects of CVB-D, as will be shown in later sections, we focused mainly on Ca_v_3.2. CVB-D at 1 μM and 3 μM exhibited stronger inhibitions of Ca_v_3.2 from a HP of −75 mV (Ca_v_3.2 under partial inactivating state) than from a HP of −100 mV (Ca_v_3.2 under resting state) (Figure 4A *p*-value of each concentration is 0.266, <0.001, 0.002, and 0.055, respectively). CVB-D has an IC_50_ of 1.47 ± 0.03 μM (Figure 4A) at HP of −75 mV, compared to that of 2.28 μM from a HP of −100 mV. Thus, CVB-D inhibition of Ca_v_3.2 is state-dependent, with a stronger blockade of inactivated channels. However, CVB-D inhibition of Ca_v_2.2 is virtually the same at a HP of −80 or −60 mV (Figure 4B *p* = 0.755). CVB-D (2.50 μM) notably shifted the half-inactivation potential (V_1/2inact_) of Ca_v_3.2 from −60.53 ± 0.47 mV to −64.19 ± 0.89 mV, as well as potently altered the slope factor of the inactivation curve from 2.57 ± 0.16 mV to 3.54 ± 0.11 mV (Figure 4C *p* = 0.001 for comparison of V_1/2inact_, *p* < 0.001 for comparison of slope factor). CVB-D showed negligible effect on the half-activation potential (V_1/2act_) of Ca_v_3.2 (−51.99 ± 0.77 mV VS. −52.91 ± 0.87 mV), but it noticeably augmented the slope factor of the activation curve from 3.48 ± 0.21 mV to 5.10 ± 0.16 mV (Figure 4D *p* = 0.447 for comparison of V_1/2act_, *p* < 0.001 for comparison of slope factor). As a result, the window current, which is represented by the overlapping area under activation and inactivation curves, was negatively shifted by CVB-D (Figure 4E). CVB-D also showed ignorable use-dependent effect on Ca_v_3.2 with similar inhibition at stimulus frequencies of 0.1 and 1 Hz, respectively (Figure 4F). Intriguingly, Z944 displays an obvious state-dependent inhibition of Ca_v_3.2 but does not affect its inactivation (Tringham et al., 2012), suggesting that these two compounds may interact with Ca_v_3.2 in different fashions.

### 3.5 Effect of cyclovirobuxine D on DRG neuron Ca_v_3.2 and Ca_v_2.2 currents and excitability

**FIGURE 5:**
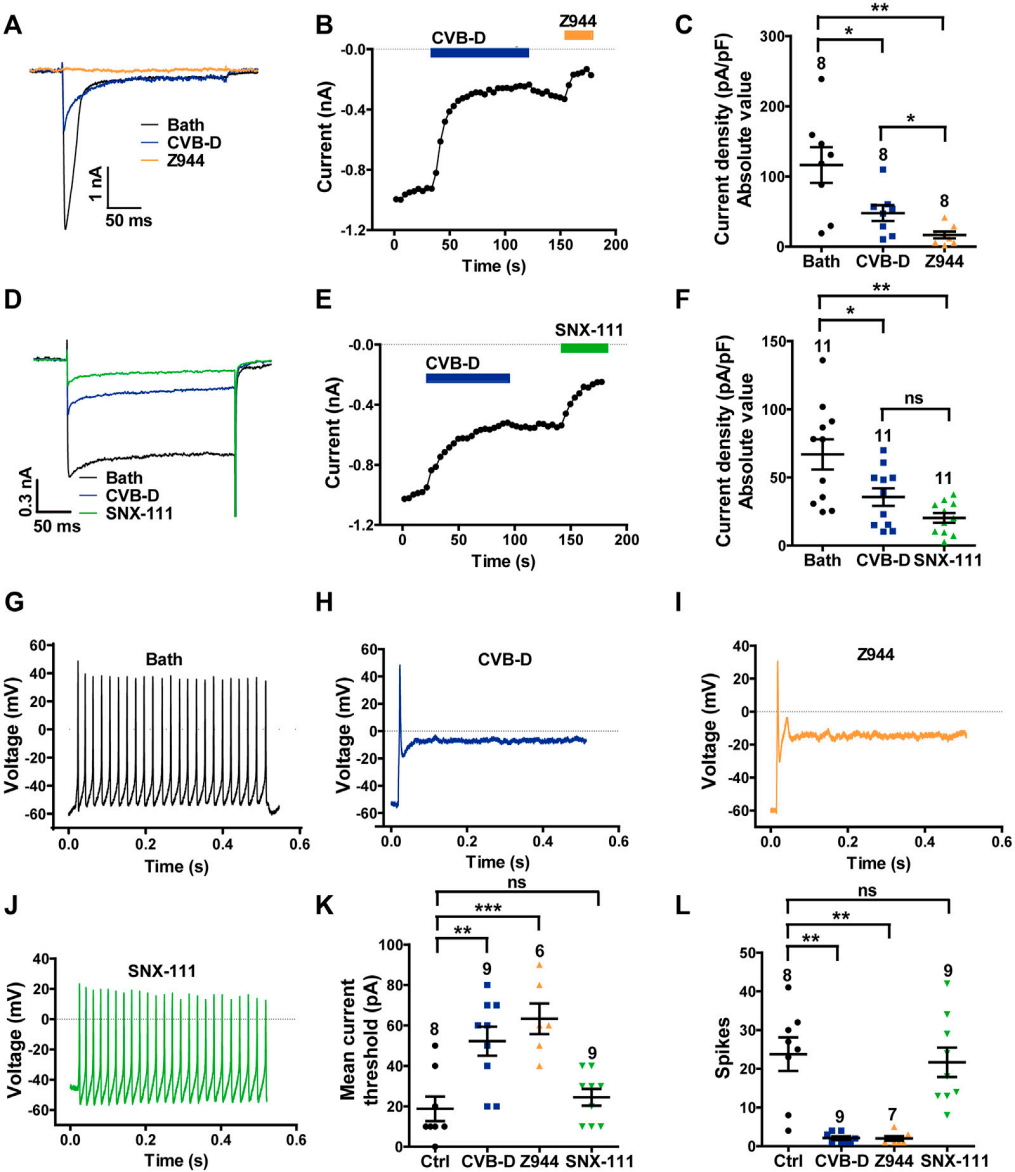
Effect of CVB-D on DRG neuron calcium channel currents and excitability. **(A)** Representative LVA (Ca_v_3.2) Ca^2+^ channel peak currents evoked by 250-ms depolarizations to -40 mV (from a HP of -100 mV) after treatment of bath (black), CVB-D (10 μM, blue) or Z944 (5 μM, orange). **(B)** Time course of inhibition by CVB-D (10 μM, blue) and subsequent inhibition by Z944 (5 μM, orange) of LVA (Ca_v_3.2) Ca^2+^ channel peak current. **(C)** Quantification of LVA (Ca_v_3.2) Ca^2+^ channel current densities from neurons treated with bath (black), CVB-D (blue) or Z944 (orange). **(D)** Representative HVA (Ca_v_2.2) Ca^2+^ channel peak currents evoked by 250-ms depolarizations to +10 mV (from a HP of -100 mV) after treatment of bath (black), CVB-D (10 μM, blue) or SNX-111 (0.5 μM, green). **(E)** Time course of inhibition by CVB-D (10 μM, blue) and subsequent inhibition by SNX-111 (0.5 μM, green) of HVA (Ca_v_2.2) Ca^2+^ channel peak current. **(F)** Quantification of HVA (Ca_v_2.2) Ca^2+^ channel current densities from neurons treated with bath (black), CVB-D (blue) or SNX-111 (green). **(G–J)** Representative recording of AP firing evoked by a threshold depolarizing current in neurons in the presence of bath **(G)**, 30 μM CVB-D **(H)**, 5 μM Z944 **(I)**, or 0.5 μM SNX-111 **(J)**. **(K)** Effect of CVB-D (blue), Z944 (orange) or SNX-111 (green) on the mean current threshold to induce AP firing in neurons. **(L)** Effect of CVB-D (blue), Z944 (orange) or SNX-111 (green) on the mean number of AP firing (spikes) in neurons evoked by a threshold depolarizing current. Data information: The number in each graph indicates the number of cells used in each experiment. Statistical significance was evaluated using two-tailed *t*-test (for two-group comparisons) or one-way analysis of variance (ANOVA) followed by Tukey’s test and LSD test (for multi-group comparisons), with *p < 0.05, **p < 0.01, and ***p < 0.001; ns indicates no significance. The exact t, F, and *p*-values are indicated in Appendix Table S1. All the data are presented as mean ± SEM.

We next examined the effect of CVB-D on native Ca_v_3.2 and Ca_v_2.2 channels, which have been shown to be the dominant nociceptive LVA and HVA calcium channels in small to medium-sized DRG neurons (diameter in 10–30 μm) (Talley et al., 1999; Rose et al., 2013; Kitano et al., 2019; Li et al., 2019; Hasan et al., 2021; Hoppanova and Lacinova, 2022). Whole-cell currents mediated by endogenous Ca_v_3.2 and Ca_v_2.2 channels were recorded from freshly dissociated mice DRG neurons using distinct depolarization voltages (Bourinet et al., 2005). Z944-sensitive Ca_v_3.2 current evoked at −40 mV was largely inhibited by 10 μM CVB-D (Figure 5A). As with recombinant Ca_v_3.2, this inhibition was relatively slow and was difficult to be reversed (Figure 5B). On average, the Ca_v_3.2 current was decreased by 58.9% by 10 μM CVB-D (Figure 5C *p* = 0.026 for Bath VS. CVB-D, *p* = 0.005 for Bath VS. Z944, and *p* = 0.022 for CVB-D VS. Z944) and was almost eliminated by 5 μM Z944 (Figure 5C). At this concentration Z944 had no effect on the HVA current evoked at +10 mV (Supplementary Figure S4A).

CVB-D (10 μM) also greatly inhibited SNX-111-sensitive, Ca_v_2.2-mediated HVA current in DRG neurons evoked at +10 mV (Figure 5D). This inhibition was slow and only slightly reversible (Figure 5E). On average, 10 μM CVB-D reduced the Ca_v_2.2 current by 46.8%, which is comparable to the effect of SNX-111 at 0.5 μM (Figure 5F *p* = 0.026 for Bath VS. CVB-D, *p* = 0.002 for Bath VS. SNX-111, and *p* = 0.056 for CVB-D VS. SNX-111). This concentration of SNX-111 did not affect Ca_v_3.2 current evoked at−40 mV (Supplementary Figure S4B).

Next, we examined the effect of CVB-D on action potential (AP) firing of DRG neurons. Freshly dissociated DRG neurons were randomly divided into four groups: control (bath solution), CVB-D (30 μM), Z944 (5 μM) and SNX-111 (0.5 μM). In control neurons, a threshold depolarizing current evoked repetitive and sustained AP firing (Figure 5G). Strikingly, a threshold depolarizing current elicited only a single spike in CVB-D-treated and Z944-treated neurons (Figures 5H,I), indicating a crucial role of Ca_v_3.2 in AP firing in these neurons. By contrast, SNX-111 did not visibly alter AP firing (Figure 5J). Compared to the control neurons, a much higher threshold depolarizing current was needed to produce an AP in CVB-D-treated and Z944-treated neurons (Figure 5K *p* = 0.003 for Ctrl VS. CVB-D, and *p* < 0.001 for Ctrl VS. Z944), and the number of AP spikes were drastically decreased (Figure 5L *p* = 0.002 for Ctrl VS. CVB-D, and *p* = 0.001 for Ctrl VS. Z944). No such effects were observed in SNX-111-treated neurons (Figure 5K, L. *p* = 0.911 for Ctrl VS. SNX-111 in Figure 5K, and *p* = 0.721 for Ctrl VS. SNX-111 in Figure 5L).

These results indicate that CVB-D potently inhibits native Ca_v_3.2 and Ca_v_2.2 channels and that inhibition of Ca_v_3.2, but not Ca_v_2.2, greatly decreases the excitability of DRG neurons.

### 3.6 Ca_v_3.2 plays a major role in the analgesic effects of cyclovirobuxine D

**FIGURE 6:**
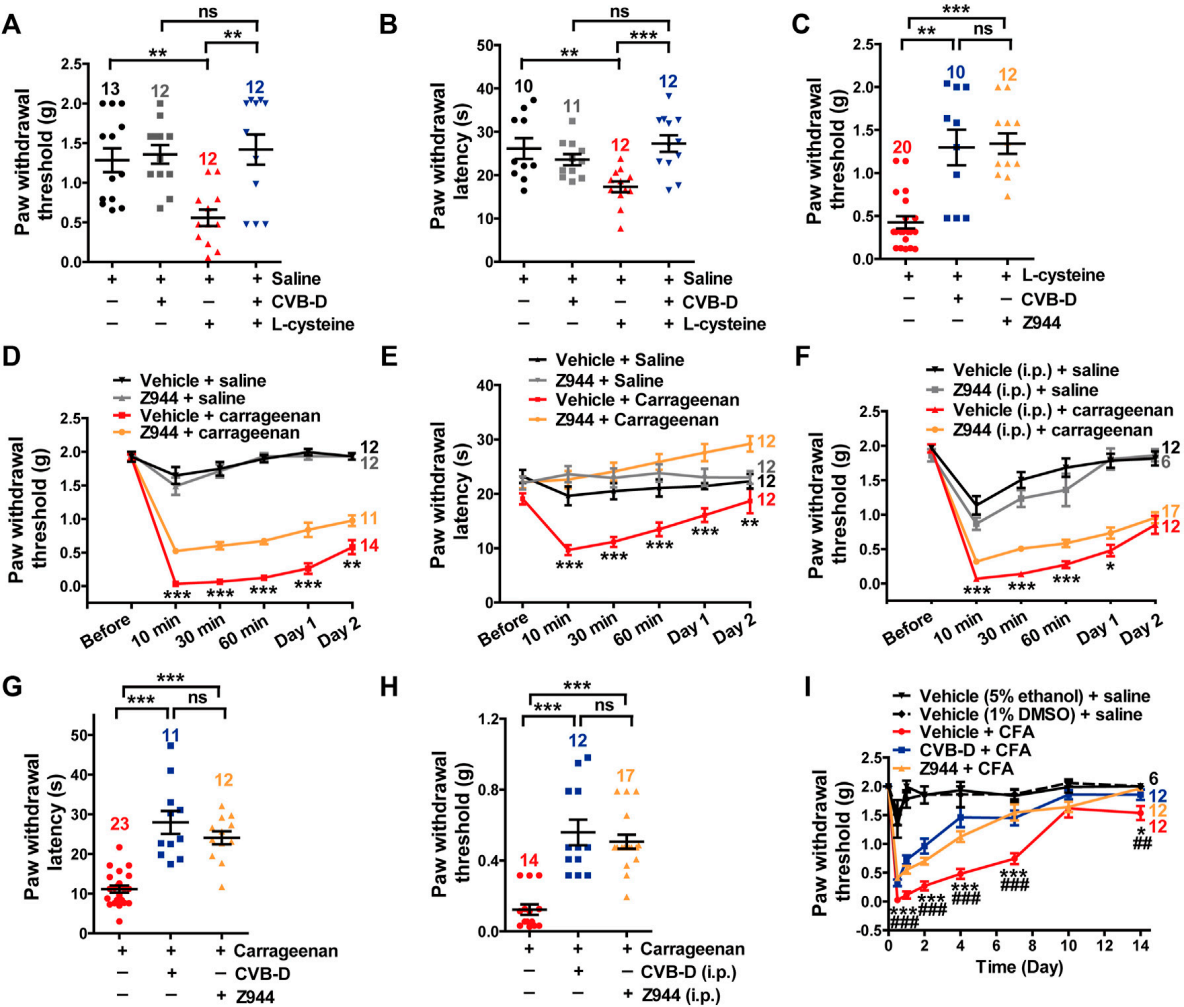
Major role of Ca_v_3.2 in the analgesic effects of CVB-D in mouse models. **(A)** Effect of CVB-D on l-cysteine-induced mechanical hypersensitivity. Saline (black) was used as a control for l-cysteine (red), and vehicle (gray) was used as a control for CVB-D (blue). PWT was measured with von Frey filaments at 10 min after i. pl. injection of saline (black), CVB-D (gray), and l-cysteine (red and blue). **(B)** Effect of CVB-D on l-cystetine-induced thermal hypersensitivity. Same grouping and experimental procedures as in A, except that PWL was measured by the hot plate assay. **(C)** Comparison of the analgesic effect of CVB-D and Z944 on l-cystetine-induced mechanical hypersensitivity. **(D)** Effect of Z944 (i.pl. injection) on carrageenan-induced mechanical hypersensitivity. Same experimental procedures as in Figure 1G, except that Z944 (3.84 μg/paw, orange) was intraplantarly injected 30 min prior to carrageenan injection (red). Vehicle-saline (black) was used as a control for vehicle-carrageenan (red). Z944-saline (gray) was used as a control for Z944-carrageenan (orange). Statistical analysis was performed between the groups of Z944-carrageenan and Vehicle-carrageenan. **(E)** Effect of Z944 (i.pl. injection) on carrageenan-induced thermal hypersensitivity. Same experimental procedures as in Figure 6D. Statistical analysis was performed between the groups of Z944-carrageenan (orange) and Vehicle-carrageenan (red). **(F)** Effect of Z944 (i.p. injection) on carrageenan-induced mechanical hypersensitivity. Same experimental procedures as in Figure 6D, except that Z944 (1 mg/kg, orange) and its corresponding vehicle (gray) were intraperitoneally injected 30 min prior to the injection of carrageenan (red). Statistical analysis was performed between the groups of Z944-carrageenan and Vehicle-carrageenan. **(G)** Comparison of the analgesic effect of CVB-D and Z944 (both i. pl. injection) on carrageenan-induced thermal hypersensitivity at 30 min after carrageenan treatment. **(H)** Comparison of the analgesic effect of CVB-D and Z944 (both i. p. injection) on carrageenan-induced mechanical hypersensitivity at 30 min after carrageenan treatment. **(I)** Comparison of the analgesic effect of CVB-D and Z944 (both i. pl. injection) on CFA-induced mechanical hypersensitivity. Statistical significance between the groups of CVB-D-CFA (blue) and Vehicle-CFA (red) was marked by asterisks, and that between the groups of Z944-CFA (orange) and Vehicle-CFA (red) was marked by pound signs. Data information: The number in each graph indicated the number of mice used in each experiment. Statistical significance was evaluated using two-tailed *t*-test (for two-group comparisons) or one-way analysis of variance (ANOVA) followed by Tukey’s and LSD test (for multi-group comparisons), with *p < 0.05, **p < 0.01, ***p < 0.001, ^#^ *p* < 0.05, ^##^ *p* < 0.01, and ^###^ *p* < 0.001; ns indicates no significance. The exact t, F, and *p*-values are indicated in Appendix Table S1. All the data are presented as mean ± SEM.

We next studied the importance of Ca_v_3.2 in CVB-D’s analgesic effects. l-cysteine promotes cutaneous mechanical and thermal hypersensitivity *via* a quick and specific way through Ca_v_3.2 channels (Todorovic et al., 2001). Administration of CVB-D (10.07 μg) by i. pl. injection greatly recovered both PWT and PWL of l-cysteine-induced hypersensitivity in mice (Figures 6A,B. In Figure 6A, *p* = 0.001 for saline VS. l-cystetine, *p* = 0.797 for saline-CVB-D VS. CVB-D-l-cysteine, and *p* = 0.007 for l-cysteine VS. CVB-D-l-cysteine. In Figure 6B, *p* = 0.006 for saline VS. l-cystetine, *p* = 0.130 for saline-CVB-D VS. CVB-D-l-cysteine, and *p* < 0.001 for l-cysteine VS. CVB-D-l-cysteine). Z944 (3.84 μg) applied by i. pl. injection showed a comparable analgesic effect on the tactile hypersensitivity as that of CVB-D (Figure 6C *p* = 0.002 for l-cysteine VS. CVB-D, *p* < 0.001 for l-cysteine VS. Z944, and *p* = 0.849 for CVB-D VS. Z944) without affecting the baseline PWT or PWL (Supplementary Figures S5A,B. In Supplementary Figure S5A, *p* = 0.969 for saline VS. vehicle, *p* = 0.464 for saline VS. Z944, *p* = 0.056 for vehicle VS. Z944. In Supplementary Figure S5B, *p* = 0.721 for saline VS. vehicle, *p* = 0.682 for saline VS. Z944, and *p* = 0.580 for vehicle VS. Z944).

In carrageenan-induced mouse models, i. pl. (3.84 μg) or i. p. (3 mg/kg) injection of Z944 alleviated both acute and sub-acute phases of thermal and mechanical hypersensitivity (Figures 6D–F *p*-value in Figure 6D of each test point is 0.848, <0.001, <0.001, <0.001, <0.001, and 0.008, respectively. *p*-value in Figure 6E of each test point is 0.055, <0.001, <0.001, <0.001, <0.001, and 0.001, respectively. *p*-value in Figure 6F of each test point is 0.627, <0.001, <0.001, <0.001, 0.039, and 0.481, respectively). These effects were comparable to those of CVB-D, as exemplified by a comparison of the effects at the testing time of 30 min (Figures 6G,H. In Figure 6G, *p* < 0.001 for carrageenan VS. CVB-D, *p* < 0.001 for carrageenan VS. Z944, and *p* = 0.247 for CVB-D VS. Z944. In Figure 6H, *p* < 0.001 for carrageenan VS. CVB-D, *p* < 0.001 for carrageenan VS. Z944, and *p* = 0.247 for CVB-D (i.p.) VS. Z944). Noticeably, as with CVB-D, a single dose of Z944 by i. pl. injection persistently eliminated mechanical hypersensitivity from day 1 to day 7 in CFA-induced mouse model (Figure 6I and Supplementary Figure S5E. In Figure 6I, *p*-value of vehicle-CFA VS. CVB-D-CFA of each test point is < 0.001, <0.001, <0.001, <0.001, <0.001, 0.185, and 0.048, respectively. *p*-value of vehicle-CFA VS. Z944-CFA of each test point is < 0.001, <0.001, <0.001, <0.001, <0.001, 0.881, and 0.005, respectively. *p*-value of CVB-D-CFA VS. Z944-CFA of each test point is 0.109, 0.081, 0.102, <0.001, 0.618, 0.097, and 0.268, respectively. In Supplementary Figure S5E, *p*-value of vehicle-CFA VS. Z944-CFA of each test point is < 0.001, <0.001, <0.001, <0.001, <0.001, 0.881, and 0.969, respectively). Z944 (i.p. injection) alone did not affect the baseline PWT (Supplementary Figure S5C. *p* = 0.508 for saline VS. vehicle, *p* = 0.633 for saline VS. Z944, and *p* = 0.431 for vehicle VS. Z944). The vehicles for CVB-D and Z944 showed negligible effects on the basal mechanical sensation and carrageenan-induced tactile hypersensitivity (Supplementary Figure S5D. *p*-value of vehicle (5% ethanol) + saline VS. vehicle (1% DMSO) + saline of each test point is 0.963, 0.123, 0.630, 0.031, 0.235, and 1.000, respectively. *p*-value of vehicle (5% ethanol) + carrageenan VS. vehicle (1% DMSO) + carrageenan of each test point is 0.604, 0.517, 0.470, 0.811, 0.155, and 0.039, respectively).

These results suggest that Ca_v_3.2 plays a major role in the analgesic effects of CVB-D as well as Z944.

### 3.7 Ca_v_2.2 plays a minor role in the analgesic effects of cyclovirobuxine D

**FIGURE 7:**
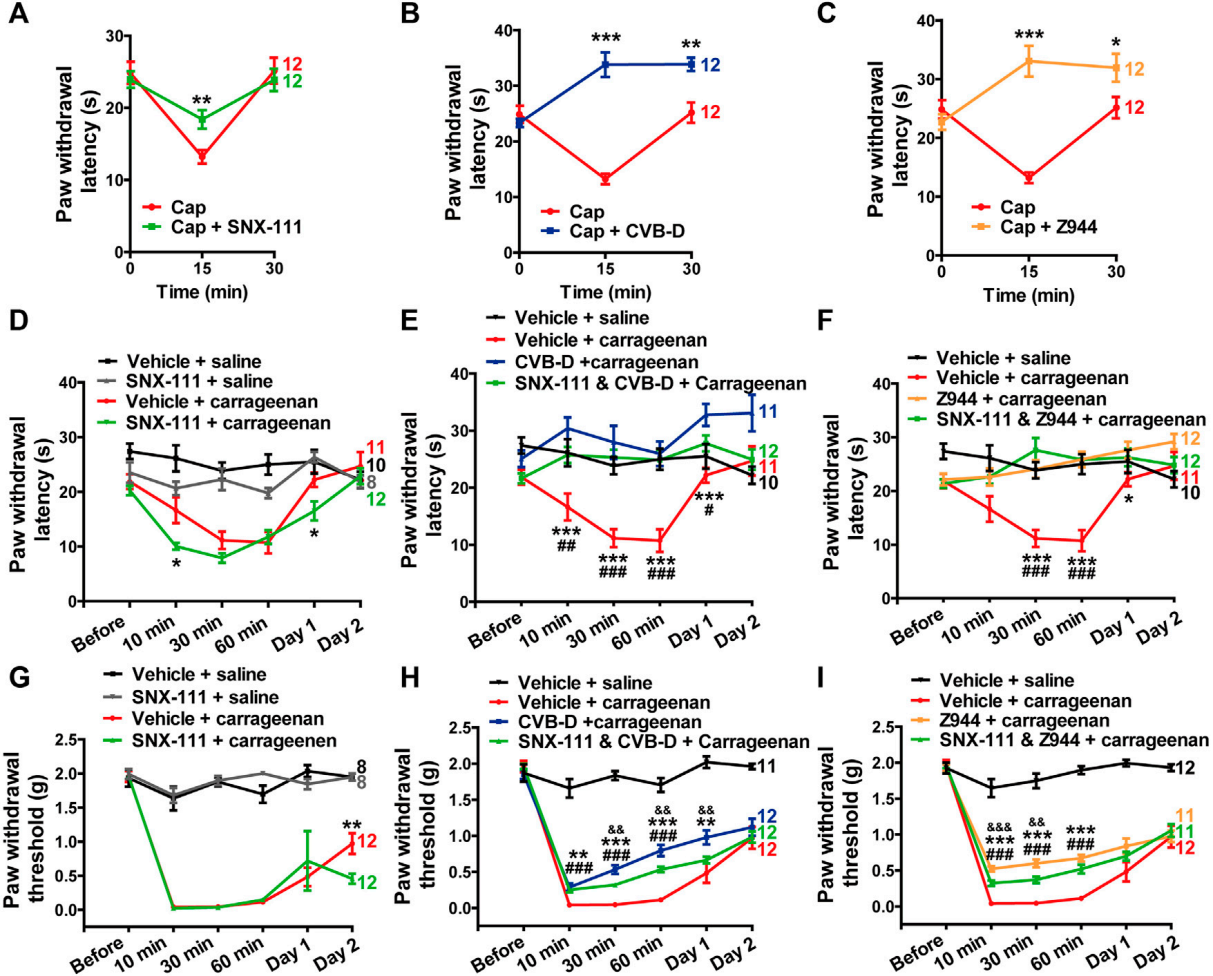
Minor role of Ca_v_2.2 in the analgesic effect of CVB-D in mouse models. **(A–C)** Effect of SNX-111 (green), CVB-D (blue), or Z944 (orange) on Cap-induced thermal hypersensitivity. PWL was measured by the hot plate assay at before (0min), and 15 and 30 min after i. pl. injection of Cap. (Red). **(D)** Effect of SNX-111 on carrageenan-induced thermal hypersensitivity. Same experimental procedures as in Figure 1E, except that SNX-111was intraplantarly injected 30 min prior to carrageenan injection. Statistical analysis was performed between the groups of SNX-111-carrageenan (green) and Vehicle-carrageenan (red). **(E,F)** Synergistic effect of SNX111 and CVB-D or Z944 on carrageenan-induced thermal hypersensitivity. Same experimental procedures as in D, except that SNX-111 and CVB-D or Z944 were intraplantarly injected together 30 min prior to carrageenan injection. Statistical significance between the groups of CVB-D-carrageenan (blue) or Z944-carrageenan (orange) and Vehicle-carrageenan (red) was marked by asterisks, and that between the groups of CVB-D-SNX-111-carrageenan (green) or Z944-SNX-111-carrageenan (green) and Vehicle-carrageenan (red) was marked by pound signs. **(G)** Effect of SNX-111 on carrageenan-induced mechanical hypersensitivity. Same experimental procedures as in Figure 1E, except that SNX-111 was intraplantarly injected 30 min prior to carrageenan injection. Statistical significance between the groups of SNX-111-carrageenan (green) and Vehicle-carrageenan (red) was marked by asterisks. **(H)** Synergistic effect of SNX111 and CVB-D on carrageenan-induced mechanical hypersensitivity. Same experimental procedures as in Figure 7G, except that SNX-111 and CVB-D were intraplantarly injected together 30 min prior to carrageenan injection. Statistical significance between the groups of CVB-D-carrageenan (blue) and Vehicle-carrageenan (red) was marked by asterisks, that between the groups of CVB-D-SNX-111-carrageenan (green) and Vehicle-carrageenan (red) was marked by pound signs, and that between the groups of CVB-D-SNX-111-Carrageenan (green) and CVB-D-Carrageenan (blue) was marked by ampersand. **(I)** Synergistic effect of SNX111 and Z944 on carrageenan-induced mechanical hypersensitivity. Same experimental procedures as in H. Statistical significance between the groups of Z944-carrageenan (orange) and Vehicle-carrageenan (red) was marked by asterisks, that between the groups of Z944-SNX-111-carrageenan (green) and Vehicle-carrageenan (red) was marked by pound signs, and that between the groups of Z944-SNX-111-carrageenan (green) and Z944-carrageenan (orange) were marked by ampersand. Data information: The number in each graph indicated the number of mice used in each experiment. Statistical significance was evaluated using two-tailed *t*-test (for two-group comparisons) or one-way analysis of variance (ANOVA) followed by Tukey’s and LSD test (for multi-group comparisons), with *p < 0.05, **p < 0.01, ***p < 0.001, ^#^ *p* < 0.05, ^##^ *p* < 0.01, ^###^ *p* < 0.001, *p* < 0.05, *p* < 0.01, and *p* < 0.001. The exact t, F, and *p*-values are indicated in Appendix Table S1. All the data are presented as mean ± SEM.

Ca_v_2.2 plays a crucial role in AP-induced transmitter release in the dorsal horn (Jevtovic-Todorovic and Todorovic, 2006; Yu et al., 2008). It has been reported that Ca_v_2.2 also participates in TRPV1-mediated thermal hypersensitivity in peripheral axons innervating the skin (DuBreuil et al., 2021). Consistent with this study, we observed that i. pl. injection of SNX-111 (53 ng) attenuated TRPV1-mediated thermal hypersensitivity (Figure 7A *p*-value of Cap VS. Cap + SNX-111 of each test point is 0.634, 0.003, and 0.598, respectively). However, CVB-D (10.07 μg) or Z944 (3.84 μg) administrated by i. pl. injection showed a much stronger analgesic effect than SNX-111 did (Figures 7B,C *p*-value of Cap VS. Cap + CVB-D f of each test point is 0.383, <0.001, and 0.001, respectively. *p*-value of Cap VS. Cap + Z944 of each test point is 0.304, <0.001, and 0.034, respectively). Indeed, the PWL at 15 or 30 min after Cap injection was even longer than the baseline PWL (0 min) (Figures 7B,C). These results suggest that Ca_v_3.2 plays a bigger role than Ca_v_2.2 does in attenuation of TRPV1-mediated thermal hypersensitivity by CVB-D. Interestingly, i. pl. injection of SNX-111 or CVB-D alone, but not Z944, increased the basal PWL (both at 15 or 30 min) (Supplementary Figure S6A–C. *p*-value of saline VS. saline + SNX-111 of each test point is 0.525, 0.021, and 0.007, respectively. *p*-value of saline VS. saline + CVB-D of each test point is 0.918, 0.040, and 0.011, respectively. *p*-value of saline VS. saline + Z944 of each test point is 0.690, 0.379, and 0.771, respectively), suggesting that Ca_v_2.2 also contributes to the analgesic effect of CVB-D on thermal hypersensitivity.

Next, we examined whether the specific Ca_v_2.2 blocker SNX-111 could alleviate inflammatory hypersensitivity as CVB-D did. SNX-111 (53 ng) administrated by i. pl. injection had negligible effect on thermal or mechanical hypersensitivity in carrageenan-induced mouse model (Figures 7D,G *p*-value of Carrageenan VS. Carrageenan + SNX-111 in Figure 7D of each test point is 0.315, 0.021, 0.079, 0.659, 0.017, and 0.537, respectively. In Figure 7G, *p* = 0.611 for Carrageenan VS. Carrageenan + SNX-111 at Day 1, and *p* = 0.008 for Carrageenan VS. Carrageenan + SNX-111 at Day 2). Furthermore, a combination of SNX-111 with CVB-D or Z944 showed ignorable synergistic analgesic effect (Figures 7E,F,H,I. In Figure 7E, *p*-value of Carrageenan VS. Carrageenan + SNX-111 and CVB-D of each test point is 0.990, 0.005, <0.001, <0.001, 0.042, and 0.997, respectively. *p*-value of Carrageenan VS. Carrageenan + CVB-D of each test point is 0.182, <0.001, <0.001, <0.001, <0.001, and 0.070, respectively. *p*-value of Carrageenan + CVB-D VS. Carrageenan + SNX-111 and CVB-D of each test point is 0.133, 0.205, 0.420, 0.915, 0.075, and 0.073, respectively. In Figure 7F, *p*-value of Carrageenan VS. Carrageenan + SNX-111 and Z944 of each test point is 0.946, 0.060, <0.001, <0.001, 0.168, and 0.998, respectively. *p*-value of Carrageenan VS. Carrageenan + Z944 f of each test point is 0.985, 0.062, <0.001, <0.001, 0.047, and 0.219, respectively. *p*-value of Carrageenan + Z944 VS. Carrageenan + SNX-111 and Z944 of each test point is 0.876, 1.000, 0.376, 1.000, 0.800, and 0.226, respectively. In Figure 7H, *p*-value of Carrageenan VS. Carrageenan + SNX-111 and CVB-D of each test point is 0.999, <0.001, <0.001, <0.001, 0.223, and 0.997, respectively. *p*-value of Carrageenan VS. Carrageenan + CVB-D of each test point is 0.468, <0.001, <0.001, <0.001, 0.009, and 0.675, respectively. *p*-value of Carrageenan + CVB-D VS. Carrageenan + SNX-111 and CVB-D of each test point is 0.456, 0.565, 0.007, 0.005, 0.007, and 0.727, respectively. In Figure 7I, *p*-value of Carrageenan VS. Carrageenan + SNX-111 and Z944 of each test point is 0.999, <0.001, <0.001, <0.001, 0.336, and 0.856, respectively. *p*-value of Carrageenan VS. Carrageenan + Z944 of each test point is 0.917, <0.001, <0.001, <0.001, 0.062, and 1.000, respectively. *p*-value of Carrageenan + Z944 VS. Carrageenan + SNX-111 and Z944 of each test point is 0.900, <0.001, 0.004, 0.065, 0.638, and 0.872, respectively).

Taken together, these results suggest that Ca_v_3.2 plays a major role and Ca_v_2.2 plays a minor role in the analgesic effects of CVB-D in mouse models.

### 3.8 Analgesic effect of cyclovirobuxine D on paclitaxel-induced peripheral neuropathy

**FIGURE 8:**
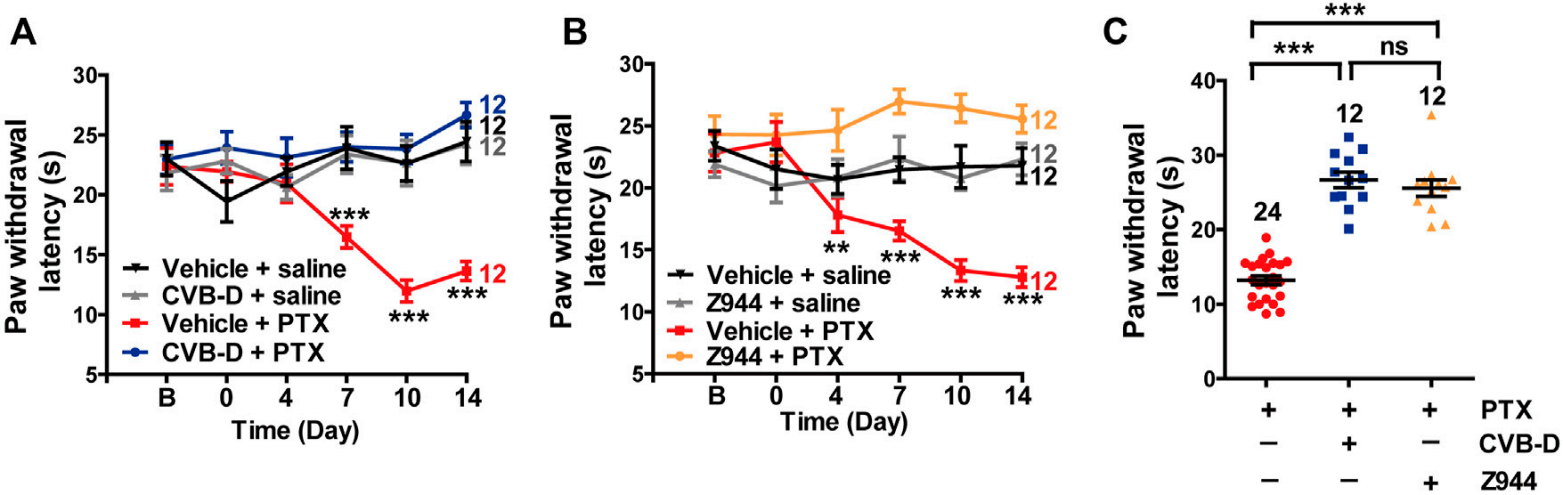
Effect of CVB-D on PTX-induced peripheral neuropathy. **(A)** Effect of CVB-D on PTX-induced thermal hypersensitivity. PWL was measured by the hot plate assay in a 2-week period. Vehicle-saline (black) was used as a control for vehicle-PTX (red), and CVB-D-saline (gray) was used as a control for CVB-D-PTX (blue). Statistical analysis was performed between the groups of CVB-D-PTX (blue) and Vehicle-PTX (red). **(B)** Effect of Z944 on PTX-induced thermal hypersensitivity. Same experimental procedures as in A, except that the mice received i. pl. injection of Z944 30 min prior to treatment with PTX. Vehicle-saline (black) was used as a control for vehicle-PTX (red), and Z944-saline (gray) was used as a control for Z944-PTX (orange). Statistical analysis was performed between the groups of Z944-PTX (orange) and Vehicle-PTX (red). **(C)** Comparison of the analgesic effect of CVB-D and Z944 on PTX-induced thermal hypersensitivity on day 14. Data information: Statistical significance was evaluated using two-tailed *t*-test (for two-group comparisons) or one-way analysis of variance (ANOVA) followed by Tukey’s and LSD test (for multi-group comparisons), with *p < 0.05, **p < 0.01, and ***p < 0.001; ns indicates no significance. The exact t, F, and *p*-values are indicated in Appendix Table S1. All the data are presented as mean ± SEM.

Paclitaxel (PTX) is a well-known anti-tumor drug worldwide (Ozols et al., 2003). However, PTX induces unbearable peripheral neuropathy, which often limits its clinical application (Ozols et al., 2003). Because Ca_v_3.2 is highly involved in PTX-induced mouse neuropathy (Li et al., 2017), we tested the analgesic effect of CVB-D on this pain model. Administration of PTX (4 mg/kg) by i. p. injection every other day for a total of four applications (days 0, 2, 4, and 6) induced severe thermal hypersensitivity, which lasted through the duration of the experiments (14 days) (Figure 8A). A single dose of CVB-D (10.07 μg) by i. pl. injection in day 0 eliminated the thermal hypersensitivity (Figure 8A *p*-value of each test point is 0.767, 0.226, 0.346, <0.001, <0.001, and <0.001, respectively). Z944 produced a comparable analgesic effect (Figures 8B,C *p*-value in Figure 8B of each test point is 0.494, 0.804, 0.005, <0.001, <0.001, and <0.001, respectively. In Figure 8C, *p* < 0.001 for PTX VS. CVB-D, *p* < 0.001 for PTX VS. Z944, and *p* = 0.687 for CVB-D VS. Z944). Given that CVB-D was also identified as a potential anti-tumor agent (Bailly and Zhang, 2020), it may simultaneously enhance the therapeutic effect of PTX and reduce the unwanted peripheral neuropathy.

## 4 Discussion

In the present study, we uncover a hitherto unknown strong analgesic effect of CVB-D against carrageenan- and CFA-induced inflammatory hypersensitivity and PTX-induced neuropathic pain in mouse models. We also find that CVB-D relieves capsaicin-induced thermal hypersensitivity and l-cysteine-mediated thermal and mechanical hypersensitivity in mice. Although CVB-D is a clinically used drug and has been found to have diverse actions in humans and animals, its molecular targets remain to be identified (Ke et al., 2016; Ao et al., 2019; Chinese Pharmacopoeia Commission, 2020; Wang et al., 2021; Zeng et al., 2021). Our study shows that CVB-D’s analgesic effects are achieved mainly through the inhibition of Ca_v_3.2, an essential target in modern analgesic drug development (Jevtovic-Todorovic and Todorovic, 2006; Cai et al., 2021; Xue et al., 2021). Remarkably, CVB-D has great selectivity against a diverse group of nociceptive ion channels distributed in primary sensory neurons, such as Na_v_1.7, Na_v_1.8, TRPV1, TPRA1, TRPM8, ASIC3, P_2_X_2_, and P_2_X_4_.

In DRG neurons, small membrane depolarizations trigger calcium influx through TTCCs to engender low-threshold calcium spikes, which in turn along with voltage-activated sodium channels elicit bursts of APs (Cheong and Shin, 2013). Ca_v_3.2, which is highly expressed in axons and somas of small- and medium-sized nociceptive DRG neurons, is crucial to the initiation and generation of AP firing (Talley et al., 1999; Todorovic and Jevtovic-Todorovic, 2013). CVB-D inhibits Ca_v_3.2 and also shifts the inactivation curve of Ca_v_3.2 to a more hyperpolarizing potential, making more channels inactivated at the resting membrane potential (Figure 4D). The resulting reduced Ca_v_3.2 current decreases the propensity of burst firing of DRG neurons (Figures 5G,I,L). Ca_v_2.2, which is abundantly distributed in nociceptive neurons of laminae I and II of the dorsal horn, is critical for A (Hoppanova and Lacinova, 2022). Consistent with this notion, we find that SNX-111 has a negligible effect on AP firing of small- and medium-sized DRG neurons (Figure 5J–L). Thus, our work suggests that Ca_v_3.2 is the predominant channel responsible for CVB-D’s regulation of AP firing of DRG neurons.

CVB-D and Z944 produces a much stronger analgesic effect than SNX-111 does in capsaicin-induced thermal hypersensitivity (Figures 7A–C). CVB-D and Z944, both by i. p. or i. pl. injection, also alleviate thermal hypersensitivity in carrageenan-, CFA-, and PTX-induced mouse models (Figures 1E,I, Figure 6E, and Figures 8A,B). By contrast, SNX-111 (i.pl. injection) has a negligible analgesic effect on carrageenan-induced thermal hypersensitivity (Figure 7D). Moreover, combining SNX-111 with CVB-D or Z944 does not produce synergistic or additional analgesia (Figures 7E,F). These results together suggest that Ca_v_3.2 is the main contributor to the analgesic effect of CVB-D on thermal hypersensitivity.

Notably, in carrageenan-, CFA- and PTX-induced mouse models, a single dose of CVB-D, as well as Z944, before applying the sensitizing reagents is sufficient to eliminate the development of thermal hypersensitivity (Figures 1E,I, Figure 6E, and Figures 8A,B). The underlying molecular mechanism of this long-lasting effect remains to be elucidated. One possibility is that it is related to a reduced calcium influx *via* TTCCs. This calcium influx has been shown to play an important role in sub-threshold secretory processes and in the spontaneous release of neurotransmitters from afferent nerve terminals in the dorsal horn (Hoppanova and Lacinova, 2022). We speculate that the calcium influx through Ca_v_3.2 may be important for the development and maintenance of thermal hypersensitivity. By inhibiting Ca_v_3.2 and shifting the inactivation curve and the window current of Ca_v_3.2 to a more negative potential (Figures 4D,F), CVB-D may hinder the above-mentioned physiological processes and thus prevent the initiation and/or maintenance of thermal hypersensitivity.

Ca_v_3.2 was recently identified as a selective marker of two major low-threshold mechanoreceptors (LTMRs), Aδ- and C-LTMRs (François et al., 2015). The presence of Ca_v_3.2 along LTMR-fiber trajectories is consistent with its key role in the transmission of low-threshold mechanical signals and in the development of mechanical hypersensitivity (François et al., 2015). The contribution of Ca_v_2.2 to mechanical hypersensitivity remains incompletely defined. For instance, pharmacological blockade of Ca_v_2.2 does not attenuate mechanical hypersensitivity in CFA-induced mice (Pitake et al., 2019). Ca_v_2.2 KO mice show a comparable mechanical sensation as WT animals do (Pitake et al., 2019; DuBreuil et al., 2021) but exhibit markedly reduced mechanical hypersensitivity following spinal nerve ligation (Saegusa et al., 2001). Our work shows that while CVB-D and Z944 have potent analgesic effects against mechanical hypersensitivity in carrageenan- and CFA-induced mouse models (Figures 1D,G,H, Figure 6D,F,I and Supplementary Figure S5E), SNX-111 does not alleviate mechanical hypersensitivity in carrageenan-induced mice (Figure 7G) and administration of SNX-111 together with CVB-D or Z944 produces negligible synergistic or additional analgesia (Figures 7H,I). Therefore, our findings suggest that Ca_v_3.2 is the major player for CVB-D’s analgesic effects on mechanical hypersensitivity.

While CVB-D is a safe and effective cardiovascular drug widely used in China, several animal experiments have reported some potential toxicities when it is administered systemically at relatively high doses or for prolonged durations (Liu et al., 1982; Zhao et al., 2011; Bailly and Zhang, 2020). In this study, we show that i. pl. application of CVB-D can produce strong and persistent analgesic effects (Figures 1D,E,G), suggesting that local/topical application of CVB-D may offer a safer way for treating certain forms of pain.

Our study shows that CVB-D effectively and long-lastingly relieves PTX-induced thermal hypersensitivity (Figure 8). This finding, in conjunction with recent reports of CVB-D’s promising *in vitro* and *in vivo* antiproliferative activities (Wu et al., 2015; Jiang et al., 2020; Zhang et al., 2020; Zhou et al., 2020; Liu et al., 2021; Zeng et al., 2021), also raises the potential of an application of CVB-D in cancer treatment and chemotherapy: a combination of CVB-D and PTX may offer a promising opportunity to concurrently enhance the anticancer effect of PTX and reduce its unwanted neuropathic symptoms.

In this study, we first demonstrate that CVB-D, a cardiovascular drug from traditional Chinese medicine *B. microphylla*, exhibits strong and persistent antinociceptive actions against several mouse models of inflammatory and neuropathic pain. Second, we demonstrate that the analgesic effects of CVB-D are mainly achieved through inhibition of Ca_v_3.2, a key target in modern analgesic drug development. Third, we uncover a potential of CVB-D to ameliorate the intractable neuropathic hypersensitivity associated with PTX chemotherapy while concurrently exerting its anticancer effects. In conclusion, we provide a convincing rationale and foundation for conducting clinical studies to repurpose CVB-D in pain management.

## Data Availability

The original contributions presented in the study are included in the article/Supplementary Material, further inquiries can be directed to the corresponding authors
