# Supplementary material for: Cyclovirobuxine D, a cardiovascular drug from traditional Chinese medicine, alleviates inflammatory and neuropathic pain mainly via inhibition of voltage-gated Cav3.2 channels
Source: Front Pharmacol. 2022 Dec 21;13:1081697. doi: 10.3389/fphar.2022.1081697 (PMC9811679; doi:10.3389/fphar.2022.1081697)
Supplement: Supplementary file 1 [file DataSheet2.docx]

Supplementary Material

## Supplementary Figure S1


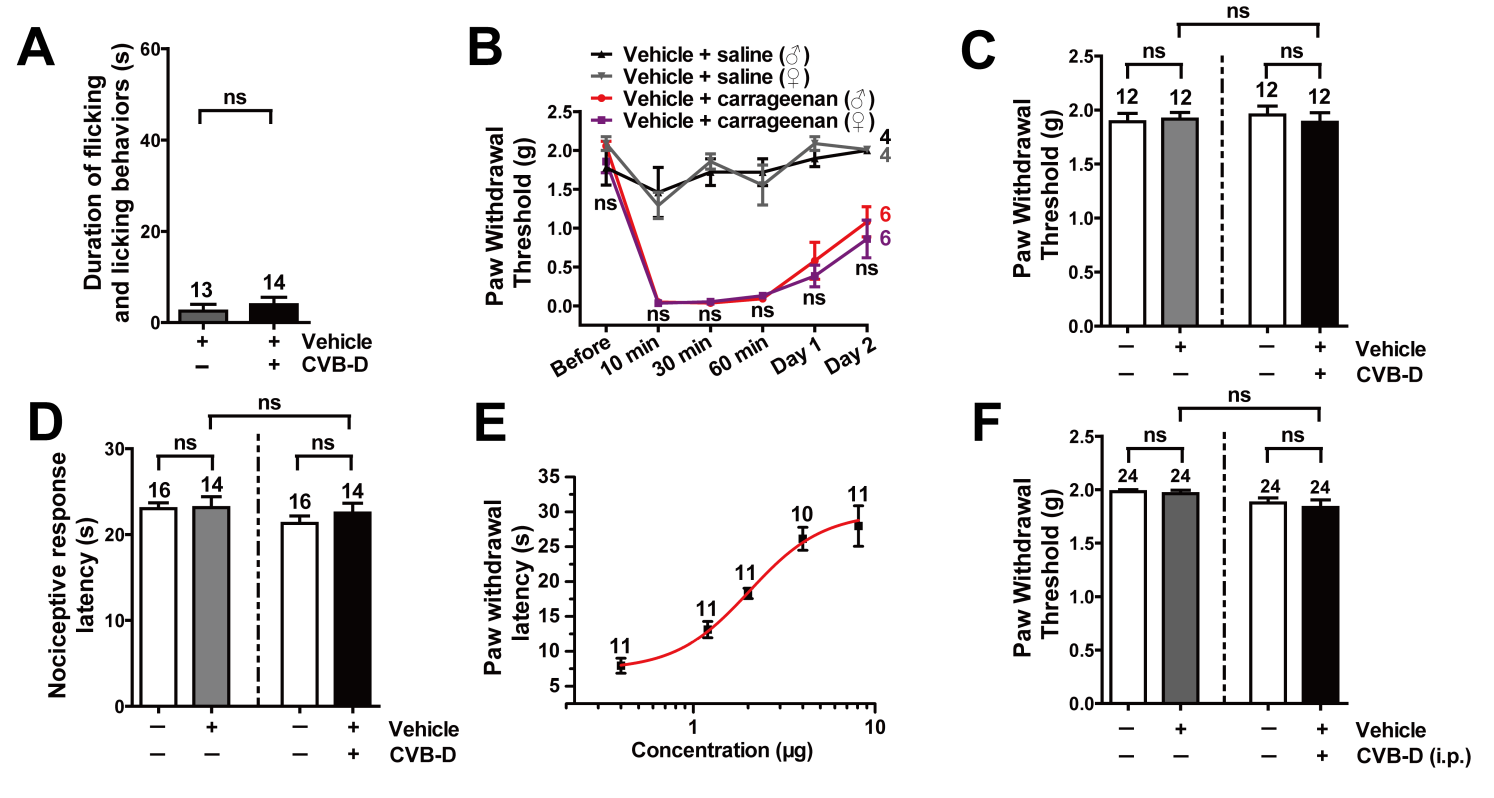


**Supplementary Figure S1. Effect of CVB-D on basal acute, mechanical and thermal nociception, and carrageenan-induced mechanical hypersensitivity in mice, as well as** **response in mice of both genders to carrageenan-induced mechanical hypersensitivity.** **(A)** Effect of vehicle or CVB-D (i.pl. injection) on basal acute nociceptive sensitivity in mice. Time of mice spent on licking or lifting of the injected hindpaws was counted within 5 min after i.pl. injection of vehicle or CVB-D. **(B)** Comparison of carrageenan-induced mechanical hypersensitivity in male and female mice. PWT was measured with von Frey filaments at the indicated times after i.pl. injection of saline or carrageenan. Vehicle and saline (i.pl. injection): male (♂), black; female (♀), gray. Vehicle and carrageenan (i.pl. injection): male (♂), red; female (♀), purple. Statistical analysis was performed between the groups of vehicle-carrageenan (♂) and vehicle-carrageenan (♀). **(C)** Effect of vehicle or CVB-D (i.pl. injection) on basal mechanical nociceptive sensitivity in mice. PWT was measured with von Frey filaments at 30 min after i.pl. injection of vehicle or CVB-D. **(D)** Effect of vehicle or CVB-D (i.pl. injection) on basal thermal nociceptive sensitivity in mice. PWL was measured using the hot plate assay at 30 min after i.pl. injection of vehicle or CVB-D. **(E)** Dose–response relationship of analgesic effect of CVB-D on carrageenan-induced mechanical hypersensitivity. Solid curve represents fit to the Hill equation. **(F)** Effect of vehicle or CVB-D (i.p. injection) on basal mechanical nociceptive sensitivity in mice. PWT was measured with von Frey filaments at 30 min after i.p. injection of vehicle or CVB-D.

Data information: The number in each graph indicates the number of mice used in each experiment. Statistical significance was evaluated using two-tailed t-test (for two-group comparisons), with ns indicates no significance. The exact t, F, P-values are indicated in Appendix Table S1. All the data are presented as mean ± SEM.

## Supplementary Figure S2


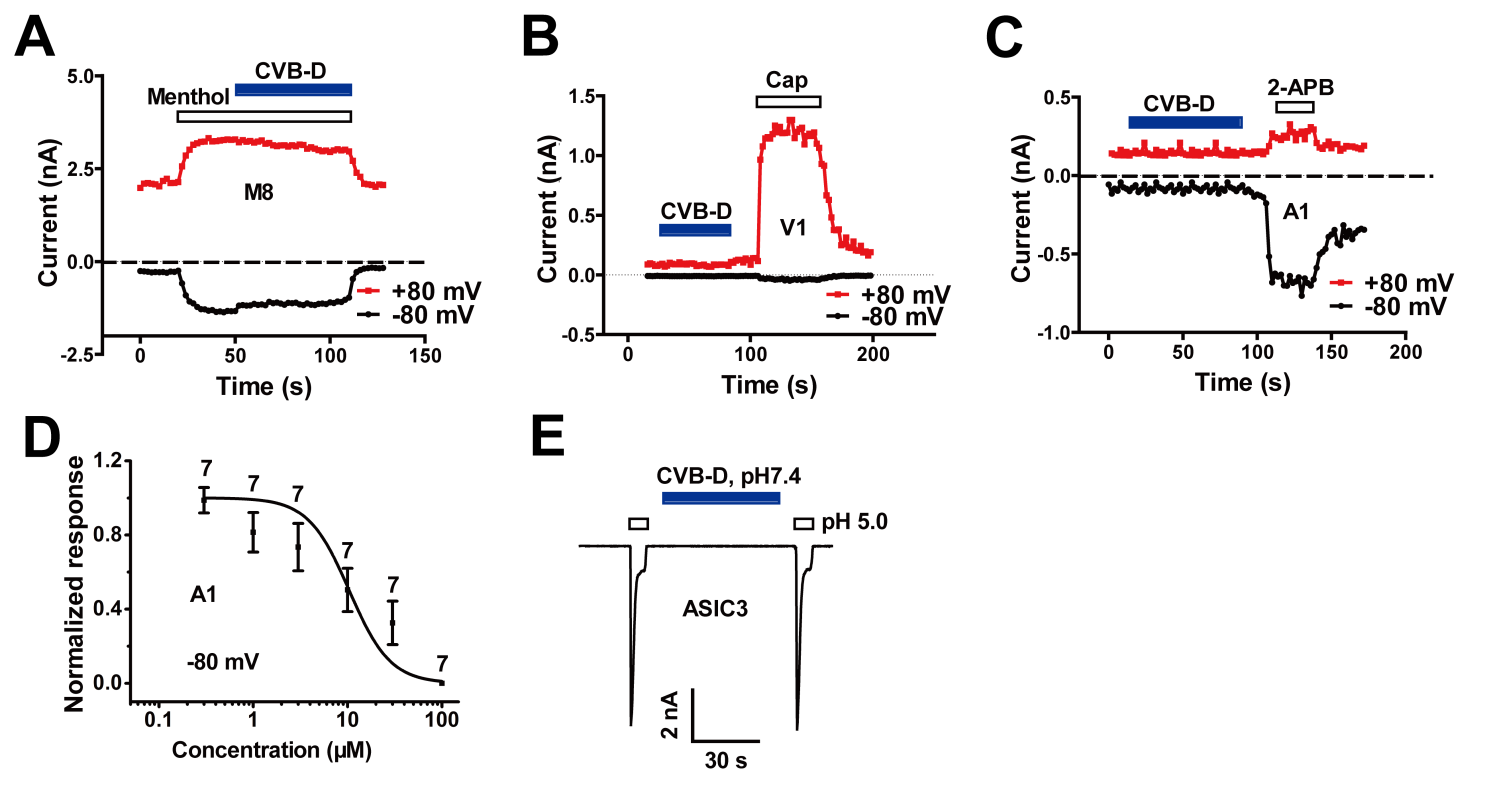


**Supplementary Figure S2. Effect of CVB-D on recombinant nociceptive ion channels.** **(A-D)** Representative whole-cell currents from HEK 293 cells expressing TRPM8 **(A)**, TRPV1 **(B)**, TRPA1 **(C)**, and ASIC3 **(E)** in the absence or presence of 30 μM CVB-D. Menthol (500 μM), Cap (1 μM), and 2-APB (200 μM) were used for activating TRPM8, TRPV1 and TRPA1, respectively. ASIC3 was activated by perfusion of extracellular solution with an acidic pH of 5.0. **(D)** Dose–response relationship of CVB-D inhibition of TRPA1 inward (-80 mV) current. Data are presented as mean ± SEM. n = 7 for each concentration. Solid curve represents fit to the Hill equation.

## Supplementary Figure S3


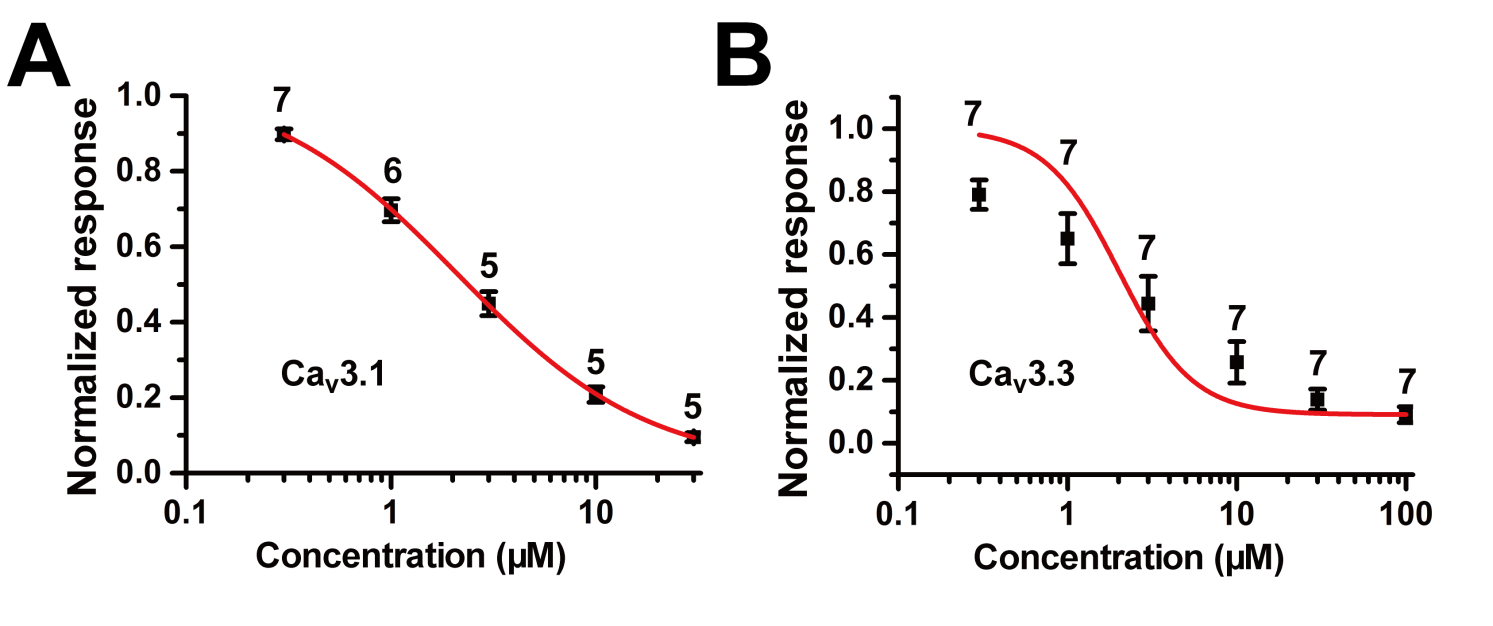


**Supplementary Figure S3. CVB-D inhibits recombinant Ca_v_3.1 and Ca_v_3.3 channels**. Dose-response relationship of CVB-D inhibition of Ca_v_3.1 **(A)** or Cav3.3 **(B)** expressed in HEK 293T cells. Data are presented as mean ± SEM. The numbers indicate the repeats of each dose. Solid curve represents fit to the Hill equation.

## Supplementary Figure S4


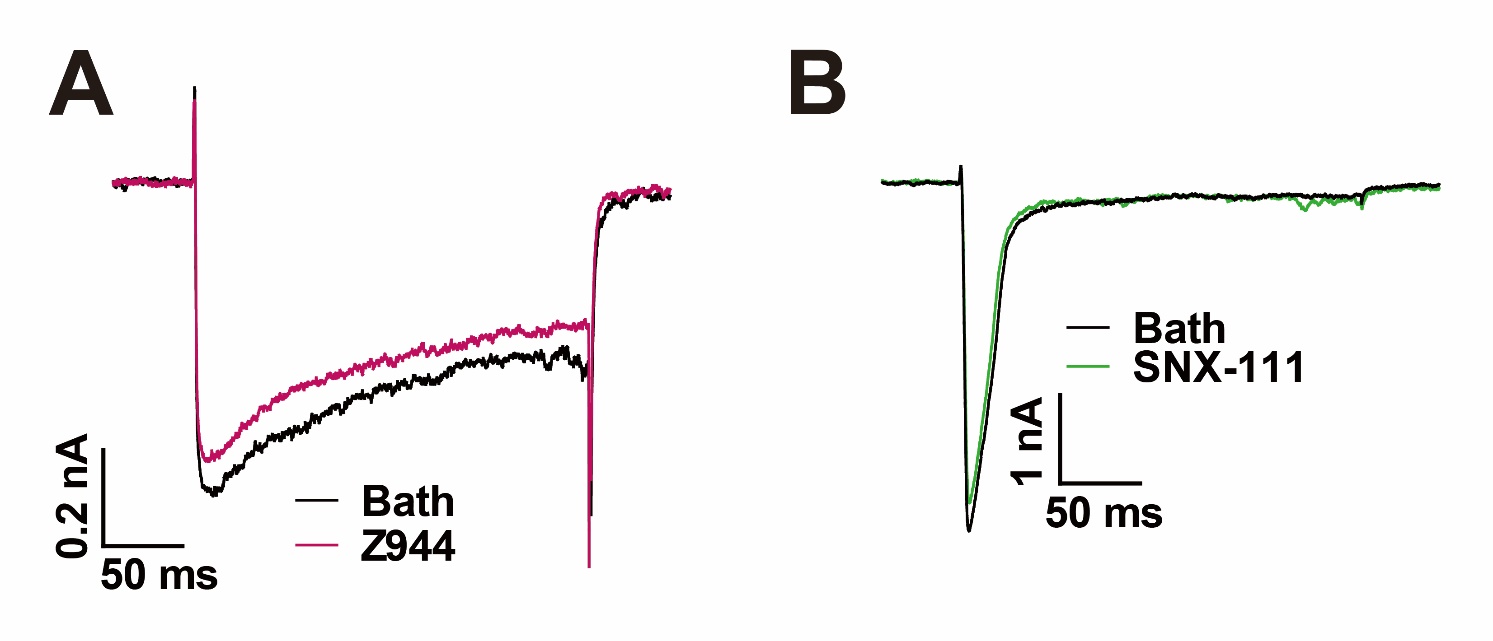


**Supplementary Figure S4. Effect of Z944 and SNX-111 on DRG neuron calcium channel currents. (A)** Representative peak current of HVA (Ca_v_2.2) Ca^2+^ channel evoked by 250 ms depolarization to +10 mV (from a HP of -100 mV) after treatment of bath solution (black) and 5 μM Z944 (pink). **(B)** Representative LVA (Ca_v_3.2) Ca^2+^ channel peak current evoked by 250 ms depolarization to -40 mV (from a HP of -100 mV) after successive treatment of bath solution (black) and 0.5 μM SNX-111 (green).

## Supplementary Figure S5


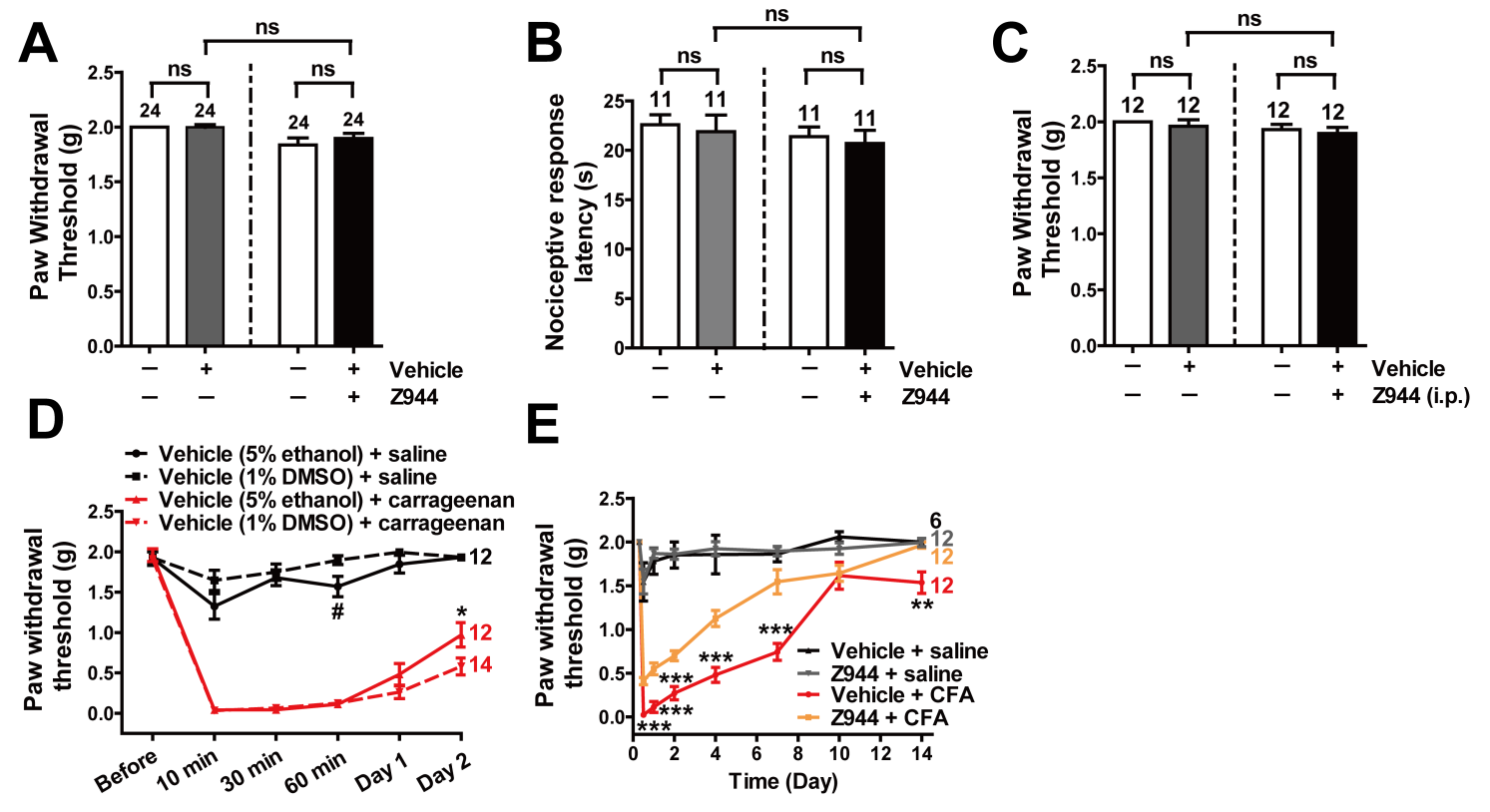


**Supplementary Figure S5. Effect of Z944 on basal mechanical and thermal nociception, and CFA-induced mechanical hypersensitivity in mice, as well as vehicle-mediated response on carrageenan-induced mechanical hypersensitivity in mice. (A)** Effect of vehicle or Z944 (i.pl. injection) on basal mechanical nociceptive sensitivity in mice. PWT was measured with von Frey filaments at 30 min after i.pl. injection of vehicle or Z944. **(B)** Effect of vehicle or Z944 (i.pl. injection) on basal thermal nociceptive sensitivity in mice. PWL was measured using the hot plate assay at 30 min after i.pl. injection of vehicle or Z944. **(C)** Effect of vehicle or Z944 (i.p. injection) on basal mechanical nociceptive sensitivity in mice. PWT was measured with von Frey filaments at 30 min after i.p. injection of vehicle or Z944. **(D)** Effect of two vehicles (i.pl. injection) on carrageenan-induced mechanical hypersensitivity. Vehicle (5% ethanol)-carrageenan and vehicle (1% DMSO)-carrageenan were showed in red line and red dash line, respectively. PWT was measured with von Frey filaments at the indicated times after i.pl. injection of saline or carrageenan. Statistical analysis between groups of Vehicle (5% ethanol)-carrageenan and vehicle (1% DMSO)-carrageenan was marked as asterisks, and between vehicle (5% ethanol)-saline and vehicle (1% DMSO)-saline was marked as pound signs. **(E)** Effect of Z944 on CFA-induced mechanical hypersensitivity. Same experimental procedures as in Fig. 1H. PWT was measured with von Frey filaments at the indicated times after i.pl. injection of saline or CFA. Statistical analysis was performed between the groups of Z944-CFA and Vehicle-CFA.

Data information: The number in each graph indicates the number of mice used in each experiment. Statistical significance was evaluated using two-tailed t-test (for two-group comparisons), with *P < 0.05; **P < 0.01, ***P < 0.001, #P < 0.05; ns indicates no significance. The exact t, F, P-values are indicated in Appendix Table S1. All the data are presented as mean ± SEM.

## Supplementary Figure S6


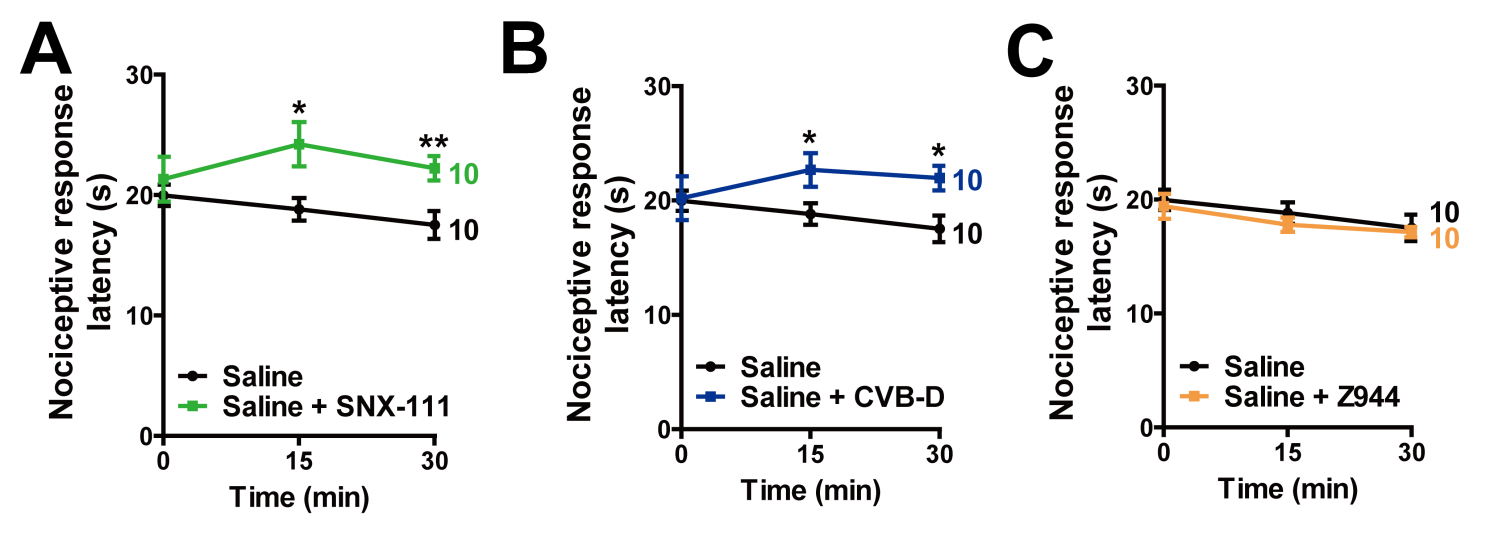


**Supplementary Figure S6. Effect of SNX-111, CVB-D or Z944 on basal thermal sensitivity in mice.** A single dose of SNX-111 (**A**, green), CVB-D (**B**, blue) or Z944 (**C**, orange) was administrated by i.pl. injection 30 min prior to the PWL measurement. PWL was recorded at before (0min), and 15 and 30 min after i.pl. injection of saline (black) using the hot plate assay.

Data information: The number in each graph indicates the number of mice used in each experiment. Statistical significance was evaluated using two-tailed t-test (for two-group comparisons), with *P < 0.05, **P < 0.01. The exact t, F, P-values are indicated in Appendix Table S1. All the data are presented as mean ± SEM.
